# Combinatorial depletions of G-protein coupled receptor kinases in immune cells identify pleiotropic and cell type-specific functions

**DOI:** 10.3389/fimmu.2022.1039803

**Published:** 2022-11-14

**Authors:** Katharina M. Glaser, Teresa K. Tarrant, Tim Lämmermann

**Affiliations:** ^1^ Max Planck Institute of Immunobiology and Epigenetics, Freiburg, Germany; ^2^ International Max Planck Research School for Immunobiology, Epigenetics and Metabolism (IMPRS-IEM), Freiburg, Germany; ^3^ Faculty of Biology, University of Freiburg, Freiburg, Germany; ^4^ Division of Rheumatology and Immunology, Department of Medicine, Duke University School of Medicine, Durham, NC, United States

**Keywords:** immune cell trafficking, leukocytes, G-protein coupled receptors, GRK, neutrophils, B cells, T cells, dendritic cells

## Abstract

G-protein coupled receptor kinases (GRKs) participate in the regulation of chemokine receptors by mediating receptor desensitization. They can be recruited to agonist-activated G-protein coupled receptors (GPCRs) and phosphorylate their intracellular parts, which eventually blocks signal propagation and often induces receptor internalization. However, there is growing evidence that GRKs can also control cellular functions beyond GPCR regulation. Immune cells commonly express two to four members of the GRK family (GRK2, GRK3, GRK5, GRK6) simultaneously, but we have very limited knowledge about their interplay in primary immune cells. In particular, we are missing comprehensive studies comparing the role of this GRK interplay for (a) multiple GPCRs within one leukocyte type, and (b) one specific GPCR between several immune cell subsets. To address this issue, we generated mouse models of single, combinatorial and complete GRK knockouts in four primary immune cell types (neutrophils, T cells, B cells and dendritic cells) and systematically addressed the functional consequences on GPCR-controlled cell migration and tissue localization. Our study shows that combinatorial depletions of GRKs have pleiotropic and cell-type specific effects in leukocytes, many of which could not be predicted. Neutrophils lacking all four GRK family members show increased chemotactic migration responses to a wide range of GPCR ligands, whereas combinatorial GRK depletions in other immune cell types lead to pro- and anti-migratory responses. Combined depletion of GRK2 and GRK6 in T cells and B cells shows distinct functional outcomes for (a) one GPCR type in different cell types, and (b) different GPCRs in one cell type. These GPCR-type and cell-type specific effects reflect in altered lymphocyte chemotaxis *in vitro* and localization *in vivo.* Lastly, we provide evidence that complete GRK deficiency impairs dendritic cell homeostasis, which unexpectedly results from defective dendritic cell differentiation and maturation *in vitro* and *in vivo*. Together, our findings demonstrate the complexity of GRK functions in immune cells, which go beyond GPCR desensitization in specific leukocyte types. Furthermore, they highlight the need for studying GRK functions in primary immune cells to address their specific roles in each leukocyte subset.

## Introduction

G-protein coupled receptor (GPCR) signaling is essential for the spatiotemporal control of leukocyte dynamics in the immune system, where several immune cell types have to coordinate their motile behavior during immune responses. Most leukocyte types express more than one GPCR on their surface, allowing them to sense a wide range of chemokines and chemoattractants in lymphoid and non-lymphoid tissues. Based on characteristic GPCR expression patterns on their cell surfaces, leukocytes of one subset can sense the same arrays of chemokines and chemoattractants. This allows them to navigate along similar routes and localize together at distinct compartments in the tissues, highlighting the essential role of GPCR signaling and mechanisms, which control GPCR functionality in the immune system (1).

G-protein coupled receptor kinases (GRKs) are a family of serine/threonine kinases with important roles for the regulation of GPCR function. Originally discovered as regulators of rhodopsin and β-adrenergic receptors in non-immune cells, the mammalian GRK family now consists of seven members (GRK1-7) that are differentially expressed in specific cell types (2). GRKs are best known for their role in homologous GPCR desensitization. Upon agonist binding and GPCR activation, GRKs are recruited to the intracellular receptor parts where they phosphorylate the C-termini. These phosphorylations recruit β-arrestins, which sterically hinder signal propagation from GPCRs to G-proteins and often initiate receptor internalization (3). By using these desensitization mechanisms, cells can terminate signaling in response to high ligand concentrations and prevent overstimulation through excessive GPCR signaling.

Immune cells also express GRKs, in particular GRK2, GRK3, GRK5 and GRK6 (4, 5). Several studies with single gene deletions of GRKs in primary immune cells have highlighted their involvement in the regulation of homologous GPCR desensitization and orchestration of leukocyte movement. As an example, GRK2 in B and T cells phosphorylates and desensitizes the spinghosine-1-phosphate (S1P)-sensing receptor S1PR1 in S1P-rich environments like the blood, which enables lymphocytes to leave the circulation and follow other chemokine gradients (6). We could recently show that GRK2 is crucial to control and self-limit neutrophil swarming behavior by desensitizing CXCR2 and LTB4R1, the receptors for the swarm-amplifying chemoattractants CXCL2 and LTB4 (7). GRK3 was reported to limit the CXCR4-mediated migration of neutrophils to CXCL12 (8) and CCR7-dependent chemotaxis of dendritic cells to CCL19 *in vitro* (9). Also, GRK6 was implicated in the control of LTB4R1, CXCR4 and CXCR2 desensitization in neutrophils (10–12). Based on these and other studies, the major role of GRKs in immune cells has been mainly assigned to the negative control of GPCR-based leukocyte motility.

However, several observations suggest additional and more variable functions of this protein family. As an example, GRK2-deficiency reduces the CCR7-dependent chemotaxis of B-cells to CCL19 (6, 13), instead of increasing it. Also, GRK6 deficiency impairs CXCR4-dependent migration of T cells to CXCL12 (14) and aspects of CCR7-dependent dendritic cell motility on haptotactic CCL21 gradients (15). These findings suggest critical roles of GRKs in the initiation rather than the termination of signaling responses through specific GPCRs. Furthermore, studies in non-immune cells reported functional interactions of GRKs with partners other than GPCRs. As an example, GRK2 can interact with and regulate many other proteins, including receptor tyrosine kinases, cytoskeletal proteins and proteins involved in cell cycle progression and cell division (16). Additionally, GRK2 can localize to unexpected cellular compartments like mitochondria and the nucleus. GRK5 even has a canonical nuclear localization sequence and was shown to act as histone deacetylase kinase, thereby indirectly regulating transcription in cardiomyocytes (17). To date, we have very limited knowledge of non-GPCR-related GRK functions in immune cells.

Our current understanding of GRKs in immune cells is largely based on studies examining leukocytes with individual GRK gene defects. This neglects that most immune cell types simultaneously express up to four GRK family members, which allows several scenarios of functional interplay between them. Only a few studies have addressed aspects of GRK specificity, redundancy and synergy in immune cells. Studies on CXCR1 and CXCR2 in a leukemic cell line showed that these receptors couple distinctly to GRK2 and GRK6 to regulate leukocyte function (11). Previous work on CCR7 expressed in non-immune HEK cells suggested a mechanism of ligand bias whereby the CCR7 ligands CCL19 and CCL21 differentially activate GRK3 and GRK6, leading to functionally different cellular outcomes (18). A more recent study exemplified how the sequential phosphorylation of one GPCR by different GRKs can produce distinct functional signaling outcomes in B cells (19). Using CXCR4 as well-defined model GPCR, it was shown that a first phosphorylation by GRK2 recruits the adaptor complex COMMD3/8, which subsequently promotes the recruitment of GRK6 to the receptor. Secondary phosphorylation by GRK6 then leads to β-arrestin recruitment and β-arrestin-dependent signaling (19). These studies strongly suggest distinct mechanisms of functional interplay between different GRKs at specific GPCRs. However, it remains unclear whether these findings from one model system can be easily extrapolated to any immune cell type, each of which shows unique expression profiles of GRK family members (5). Currently, there is a profound lack of comprehensive studies that compare the role of different GRKs (a) for multiple GPCRs within one leukocyte type, and (b) for one specific GPCR between several primary immune cell types. In addition, we completely miss a functional assessment of (c) how the depletion of more than one GRK affects immune cell function and their fate in physiological tissues.

Here, we systematically addressed the functional consequences of single, combinatorial and complete genetic depletions of GRKs on the GPCR-controlled migration and tissue localization of four primary immune cell types: neutrophils, T cells, B cells and dendritic cells (DCs). Our study provides a comprehensive comparison of GRK functions between different immune cell types.

## Materials and methods

### Mouse models

Mice were bred and maintained in a conventional animal facility at the Max Planck Institute of Immunobiology and Epigenetics (Freiburg, Germany) according to local regulations. Animal breeding was conducted with approval by the Regierungspräsidium Freiburg. All mouse strains used in this study were without health burden. Adult mice (>8 weeks) were used. WT controls were Cre-negative and age- and sex-matched for all experiments. Littermate controls were used for *ex vivo* migration experiments and analysis of endogenous immune cell populations. Removal of organs from CO_2_ euthanized mice was performed by FELASA B-trained personnel in accordance with the relevant guidelines and regulations approved by the animal welfare review committee of the MPI-IE. No animal experimentation was performed in this study. The following mouse strains were on a C57BL6/J background and have previously been generated and published: *Grk2* ^fl/fl^, (JAX stock #012458) (20), *Grk3* ^−/−^, (kindly provided by Teresa Tarrant) (21), *Grk5* ^fl/fl^ (JAX stock #010960) (22), *Grk6* ^fl/fl^ (JAX stock #010962) (23), *Mrp8*-Cre (JAX stock #021614) (24), *Vav1*-iCre (JAX stock #008610) (25), *Cd11c*-Cre (JAX stock #008068) (26), *Cd19^Cre^* (JAX stock #006785) (27), *Rorc*-Cre (JAX stock #022791) (28). Mouse crosses are indicated in the respective figures.

### Neutrophil and lymphocyte isolation

Neutrophils were isolated from bone marrow (tibia, femur, and os coxae) using the MACS neutrophil isolation kit for negative selection (Miltenyi Biotec) and an autoMACS^®^ Pro Selector cell separator (Miltenyi Biotec) according to the manufacturer’s protocol and as previously described (7). T- and B-cells were isolated from spleens by negative selection using the EasySep™ mouse T cell or EasySep™ mouse B cell isolation kit (Stemcell Technologies), respectively.

### GM-CSF culture of bone marrow-derived dendritic cells

BMDCs were generated from bone marrow (isolated from femur, tibia and os coxae) in R10 culture medium (RPMI 1640 Medium, GlutaMAX™ Supplement (Thermo Fisher Scientific), 10 U/ml penicillin (Sigma), 10 µg/ml streptomycin (Sigma), 10% heat-inactivated fetal bovine serum (Sigma)) supplemented with 20 ng/ml recombinant murine GM-CSF (PeproTech). Cultures were maintained at 37° C and 5% CO_2_ in a humidified incubator for 8-9 days. For BMDC stimulation, cells were cultured for 24 h in R10 supplemented with 20 ng/ml GM-CSF and 20 ng/ml LPS (from E.coliO127:B8, L45116, Sigma).

### Flt3-Ligand culture of bone marrow-derived dendritic cells

To generate Flt3-Ligand (FLT3L) dendritic cells, bone marrow was cultured for 8 days undisturbed in R10 culture medium supplemented with 55 μM 2-mercaptoethanol and 200 ng/ml recombinant murine FLT3L (PeproTech).

### Leukocyte labeling and under-agarose chemotaxis assay

Neutrophils used for under-agarose migration assays were labeled for 25 min with CellTracker™ Green (CMFDA, 0.5 µM) or 5-carboxy-tetramethylrhodamine, succinimidyl ester (5-TAMRA SE, 10 µM) in PBS with 0.0002% Pluronic™ F-127 (all Thermo Fisher). BMDCs were labeled for 25 min with CellTracker™ Green (CMFDA, 1 µM) or 5-carboxytetramethylrhodamine, succinimidyl ester (5-TAMRA SE, 10 µM) in PBS with 0.0002% Pluronic™ F-127. The under-agarose chemotaxis assay was performed as described elsewhere (7, 29). In brief, 1.2% (w/v) (for neutrophils) or 0.6% (w/v) (for BMDCs) ultrapure agarose (Invitrogen) in a solution of phenolred-free RPMI, 2x HBSS (both Thermo Fisher Scientific), and 10% FCS was cast into tissue culture dishes and was allowed to solidify. A central attractor hole surrounded by four responder holes in a distance of 3 mm were punched into the polymerized gel and the gel was allowed to equilibrate for one hour at 37° C and 5% CO_2_. The responder holes were loaded with differentially dye-labeled cells at a concentration of 5 × 10^6^ cells/ml (for neutrophils) or 1.25 × 10^6^ cells/ml (for BMDCs) in R10. The central well was loaded with chemokines at the indicated concentrations: LTB4 (Cayman), murine CXCL2 (PeproTech), WKYMVM (Tocris), WKYMVm (Tocris), murine C5a (PeproTech) for neutrophil chemotaxis experiments and murine CCL19 (PeproTech) for BMDC chemotaxis. In experiments with WKYMVm, neutrophils were 30 min preincubated with the FPR2 inhibitor WRW4 (Tocris, 100 µM) and also migrated in the presence of WRW4. Neutrophils were allowed to migrate for 4 h, BMDC for 16 h at 37° C and 5% CO_2_ in a humidified incubator. Live-cell video microscopy was done with a spinning-disk confocal microscope (Cell Observer SD system, Carl Zeiss, with a CSU-X1 confocal scanner unit, Yokogawa and an AxioObserver Z1 inverted microscope stand) equipped with a stage-top incubator (Tokai-Hit). Images were taken using an Evolve^®^ back-illuminated EM-CCD camera (Teledyne Photometrics) and a plan-apochromat 10x 0.45 objective. End-point images were taken at the beforementioned spinning-disk confocal microscope or a LSM780 fluorescence confocal microscope (Zeiss). Tiles were stitched using ZEN blue software (Carl Zeiss Microscopy). For analysis of migration videos, cells were manually tracked using Imaris software (versions 7.5 – 9.5, Bitplane), coordinates were exported and further analyzed using a custom R script [R version 4.0.2, RStudio version 1.3.959, ggplot2 version 3.3.2 (7)]. For the analysis of endpoint pictures, cell center points were identified using the Imaris spot function. Displacement was measured from the border of the responder hole and the median per well was calculated as one technical replicate. In rare cases, technical replicates were excluded from analysis when cells did not respond or the agarose gel was damaged.

### Transwell migration assay for lymphocytes

Transwell assays were used to analyze T-cell and B-cell migration towards various chemoattractants and chemokines. Lymphocytes isolated from spleen were incubated in serum-free medium (2% fatty-acid free BSA, 10 mM HEPES in RPMI) for 1 h at 37°C and 5% CO_2_ in a humidified incubator prior to migration. HTS Transwell^®^ 96 well plates with polycarbonate membrane and 5-µm pores were used (Corning). The lower chamber was filled with chemoattractants in serum-free medium (CXCL12, CXCL13, CCL19, CCL21, S1P; concentrations as indicated in the graphs), and cells (400.000 cells/well in serum-free medium) were placed in the upper chamber and allowed to migrate for 3 h at 37° C and 5% CO_2_ in a humidified incubator. Cells in the bottom chamber were harvested, stained with DAPI and counted using a LSRFortessa™ (BD) flow cytometer. Migration efficiency of DAPI-negative cells to all chemokines was calculated as % of input, after the rate of spontaneous migration was subtracted. As migration of WT lymphocytes to S1P was in the range of spontaneous migration, it was not subtracted in these experiments. All assays were performed in technical duplicates. In rare cases, experiments were excluded from analysis when WT cells did not show any migration.

### B cell adhesion assay

To test B cell adhesion, splenocytes were isolated and erythrocyte lysis performed. Follicular B cells (Lin^-^, CD45^+^, B220^+^, CD23^+^) and marginal zone B cells (Lin^-^, CD45^+^, B220^+^, CD21^+^, CD23^-^) were sorted on a FACSymphony™ S6 or FACSAria™ Fusion flow cytometer (BD). Angiogenesis µ-slides (Ibidi) were coated overnight with 2 µg/ml recombinant mouse ICAM-1 Fc chimera protein or 2 µg/ml recombinant mouse VCAM-1 Fc chimera protein (R&D Systems). 15.000 sorted cells were used per well, stimulated with 1 µg/ml CXCL12 and 1 µg/ml CXCL13, and incubated for 30 min at 37° C and 5% CO_2_ in a humidified incubator. Wells were carefully washed three times, Hoechst was added and complete wells were imaged using a LSM780 fluorescence confocal microscope (Zeiss). Tiles were stitched using ZEN blue software (Zeiss) and cells were counted using the Imaris spot function (Bitplane).

### DC progenitor sorting and culture

In order to obtain GM-DC and GM-Mac precursors from bone marrow (isolated from tibia, femur, os coxae), lineage-negative cells were enriched using a FITC positive selection kit (Stemcell Technologies) and FITC-conjugated antibodies against Ly6G, B220, CD3 and MHCII (all Biolegend) according to the manufacturer’s instruction. Cells were sorted on a FACSymphony™ S6 (BD) and progenitors were defined as follows: MDPs as Lineage^−^ (Ly6G, CD19, CD3, NK1.1, B220, Ter119, CD11c, MHCII, Zombie NIR™), CD115^+^, CD117^hi^, CD11b^−^, DNGR-1^−^, Ly6C^−^, CD135^+^. cMoPs as Lineage^−^, CD115^+^, CD117^hi^, CD11b^−^, DNGR-1^−^, CD135^−^, Ly6C^+^. CDPs as Lineage^−^, CD115^+^, CD117^low-neg^, CD135^+^, DNGR-1^+^ (antibodies listed in **Supplementary Table 1**) (30). For GM-CSF cultures, 10.000 sorted progenitors were mixed with 750.000 CD45.1 bone marrow cells, which are a source of growth factors for the sorted progenitors and were used as internal control of culture conditions, and cultured as described above (30).

### Flow cytometry

For flow cytometric analysis of lymphocytes from lymph nodes and spleens, cell suspensions were obtained using a 70-µm cell strainer. Erythrocyte lysis was performed with ACK lysing buffer (ThermoFisher). For the analysis of endogenous dendritic cells, spleens and lymph nodes were digested for 20 min, ear skin for 1 h at 37°C shaking at 1000 rpm in digestion buffer (0.5 mg/ml collagenase IV (abnova), 0.25 U/ml dispase (StemCell), 40 µg/ml DNase I (Roche), 10 µM MgCl_2_, 5% FCS and 1% penicillin/streptomycin in HBSS). Cultured BMDCs were harvested through gentle washing with PBS. Unspecific binding was blocked with anti-CD16/32 (BD) and cells stained for 1.5 h on ice (antibodies listed in **Supplementary Table 1**). For intracellular staining, the Foxp3/transcription factor staining buffer set (eBioscience) was used according to the manufacturer’s instructions. DAPI, Zombie NIR™ (BioLegend) or fixable viability dye eFluor 506 (eBioscience) were used as live/dead cell dyes. Data was acquired on a LSRFortessa™ (BD) and analyzed using FlowJo™ software (BD, version 10).

Gating strategies for bone marrow precursors in **Supplementary Figures 3A, B** were adapted from (31): CMPs/MDPs were defined as Lin^−^ (CD3, B220, CD19, NK1.1, Ly6G, Ter119, CD11c, CD11b), Sca-1^−^, CD117^+^, CD16/32^lo^, CD34^+^, CD135^+^; GMPs as Lin^−^, Sca-1^−^, CD117^+^, CD16/32^hi^, CD34^+^, CD135^−^, Ly6C^-^; cMoPs and GPs as Lin^−^, Sca-1^−^, CD117^+^, CD16/32^hi^, CD34^+^, CD135^−^, Ly6C^+^; BM monocytes as Lin^−^, Sca-1^−^, CD117^−^, CD16/32^hi^, Ly6C^+^; and CDPs as Lin^−^, Sca-1^−^, CD117^lo^, CD16/32^lo^, CD34^+^, CD135^+^, Ly6C^−^ (antibodies listed in **Supplementary Table 1**).

### Immunoblot analysis

Efficiencies of conditional GRK depletion in BMDCs were determined by immunoblot analysis. BMDCs were lysed (50 mM Tris, 150 mM NaCl, 5 mM EGTA, 5 mM EDTA, 0.5% IGEPAL^®^ CA-630, 1% Triton™ X-100, 1x cOmplete™ protease inhibitor cocktail (Roche)) and lysates loaded on 12% polyacrylamide gels for electrophoresis under reducing conditions (SDS-PAGE). Separated proteins were immunoblotted on PVDF membrane and unspecific binding blocked with 5% milk powder in Tris-buffered saline with 0.1% Tween^®^ 20 (TBS-T). Membranes were stained overnight at 4°C with primary antibodies (listed in **Supplementary Table 1**) and actin as loading control and, after three wash steps with TBS-T, stained with horseradish peroxidase-coupled secondary antibodies (Dako). Chemiluminescence was detected using Clarity™ Western ECL substrate and a ChemiDoc™ Imaging System (both BioRad).

### RT-qPCR

Conditional knockout efficiency of GRKs in T- and B-cells was determined by RT-qPCR. RNA was extracted from isolated splenic T- or B-cells using TRI Reagent^®^ (Sigma-Aldrich) and chloroform extraction. Reverse transcription was performed with SuperScript IV™ reverse transcriptase and random hexamere primers (ThermoFisher). qPCR was performed using ABsolute QPCR Mix, SYBR Green, ROX (ThermoFisher) according to the manufacturer’s instructions and a StepOnePlus™ Real-Time PCR system (Applied Biosystems). Primers are listed in **Supplementary Table 2**, *Grk* expression levels were normalized against *Actin* and *Gapdh*.

### Tissue processing and immunofluorescence analysis

For immunofluorescence analysis of lymphoid organs, lymph nodes were fixed with 1% PFA overnight, incubated in 30% sucrose for 8 h, and embedded in Tissue-Tek^®^ O.C.T.™ Compound (Sakura). Spleens were snap-frozen in liquid nitrogen before embedding. A CM3050 S Cryostat (Leica) was used to cut 20 µm thick sections. Before staining, spleen sections were fixed in ice-cold methanol for 10 min. Unspecific binding was blocked in blocking/staining buffer (0.1% Triton™ X-100, 1% BSA in PBS). Staining was done overnight in a humidified chamber at 4°C, antibodies are listed in **Supplementary Table 1**. Sections were mounted with Fluoromount-G™ (SouthernBiotech) and images acquired using a LSM780 fluorescent confocal microscope (Zeiss).

### Crawl-out assay and analysis

Ears were mechanically split into two halves in a way that the cartilage stayed on the dorsal side. The ventral half was placed in pre-warmed R10 medium, floating with the open, dermal side facing the liquid and incubated for 16 h at 37°C and 5% CO_2_ in a humidified incubator. After incubation, the tissue was fixed overnight in 1% PFA and subsequently subjected to immunofluorescent staining as described above using anti-MHCII antibodies for DCs and anti-Lyve1 for lymphatic vessels (see **Supplementary Table 1**). Images were acquired with a LSM780 fluorescence confocal microscope (Zeiss) using the tile function and z-stacks. Tiles were stitched with ZEN blue software (Carl Zeiss Microscopy). For image analysis, the DC signal was quantified using the surface function of Imaris (Bitplane, versions 7.5 to 9.5) to mask lymphatic vessels. In rare cases, images were excluded from analysis when the density of lymphatics in the imaging field of view clearly deviated from all other images.

### RNA sequencing

RNA was isolated from BMDCs (unstimulated or LPS-stimulated for 24h) by standard phenol-chloroform extraction in technical triplicates. Libraries were prepared using the TruSeq stranded mRNA protocol (Illumina) and sequenced on a NovaSeq6000 (Illumina) with a reading depth of 15 × 10^6^ reads per sample. RNA-seq analysis was performed using the mRNA-seq function in snakePipes (version 2.1.0) (32). In brief, raw fastq files were aligned to the mm10 reference genome using STAR (version 2.7.3a) (33) and expression count quantification was performed with featureCounts (version 2.0.0) (34). Quality control was performed using deeptools (version 3.3.2) (35). Differential gene expression analysis was performed using DESeq2 (version 1.26.0) (36) implemented in R (version 3.6.2). Expression data was further analyzed in R (R version 4.0.2, RStudio version 1.3.959). Results with a false discovery rate < 0.05 were considered significant.

### Statistical analysis

Analyses were performed using Prism software (GraphPad Software, Inc. Version 9.1.0). First, normality of sample distribution was assessed. For comparison of two groups with normally distributed data, student’s *t* test was used. For comparison of two groups with not-normally distributed data, the nonparametric Mann-Whitney test was used. Ordinary one-way ANOVA and Tukey’s *post-hoc* tests for multiple comparisons were used for comparison of more than two groups of normally distributed data. Kruskal-Wallis tests and Dunn’s *post-hoc* tests were used for comparison of more than two groups of not-normally distributed data. For ratio datasets, log2 transformed values were computed and one sample *t* tests with a theoretical mean = 0 were used. “ns” indicates non-significant difference (*P*>0.05), stars indicate significance (*P<0.05, **P<0.01, ***P<0.001). For further statistical details, see Supplementary Table 3.

## Results

### Neutrophils lacking all four GRKs show a general increase in chemotactic responses

**Figure 1:**
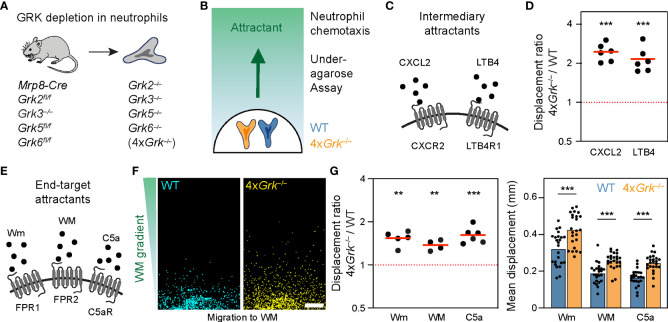
Neutrophils lacking all four GRKs show a general increase in chemotactic responses **(A)** Scheme of the mouse model used for the generation and isolation of neutrophils with complete lack of all four GRK isoforms (see (7) for gene knockout efficiency). **(B)** Scheme of the under-agarose chemotaxis assay, in which differentially stained WT and 4x*Grk* ^−/−^ neutrophils migrate side by side towards a chemoattractant gradient. **(C)** Scheme of intermediary attractants and their corresponding GPCRs CXCR2 and LTB4R1 expressed by neutrophils. **(D)** Migration to 1 µM CXCL2 and 1 µM LTB4 was measured after 4 h and is displayed as displacement ratios of 4x*Grk* ^–/–^ versus WT cells. *n* = 6 biological replicates performed as independent experiments with *N* = 3-8 technical replicates each. Bars display the mean. ***P < 0.001, **P < 0.01, one sample *t* test. **(E)** Scheme of end-target attractant stimuli and their corresponding GPCRs FPR1, FPR2 and C5aR expressed by neutrophils. **(F)** Migration to 50 µM WM peptide, endpoint images after 4 h. Scale bar: 200 µm. **(G)** Migration to 1 µM Wm, 50 µM WM and 1 µM C5a was measured after 4 h and is displayed as displacement ratios of 4x*Grk* ^–/–^ versus WT (left) and mean displacement (right). *n* = 4-6 biological replicates performed as independent experiments with *N* = 3-8 technical replicates each. Bars display the mean. ***P < 0.001, **P < 0.01, one sample *t* test (left), *t* test (right).

We began our study by examining neutrophils, which are key cells of the innate immune response with crucial roles for clearing invading bacteria and fungi in tissues. Neutrophils express a diverse repertoire of many GPCRs on their surface, allowing them to respond and migrate to a wide range of danger-associated signals, inflammatory chemokines and chemoattractants (37). In a previous study we carefully characterized the functional role of GRK2 for the desensitization of LTB4R1 and CXCR2, the two important GPCRs that mediate neutrophil swarming (7). We analyzed neutrophils responding to gradients of the swarm attractants LTB4 and CXCL2 in an under-agarose chemotaxis assay, and demonstrated that *Grk2*-deficient neutrophils show twice the displacement of control cells and continue to migrate in areas of high attractant concentrations. However, *Grk2*-deficient neutrophils did not show this enhanced migration in gradients of attractants binding to formyl peptide receptors (FPR1 and FPR2) or the complement component 5a anaphylatoxin chemotactic receptor 1 (C5aR1) (7). In that study, we also described *Mrp8-Cre Grk2^fl/fl^ Grk3^−/−^ Grk5^fl/fl^ Grk6^fl/fl^* mice, which we used to generate and characterize primary mouse neutrophils with a complete depletion of all four expressed GRKs (referred to as 4x*Grk* ^−/−^) (**Figure 1A**) (7). These cells showed the same migratory behavior as *Grk2* ^−/−^ neutrophils in combined gradients of LTB4 and CXCL2, highlighting the particular role of GRK2 in controlling the swarm-mediating receptors LTB4R1 and CXCR2 (7). Here, we expanded our analysis of 4x*Grk* ^−/−^ neutrophils and investigate their chemotactic responses toward a more diverse set of attractants. Wildtype (WT) and 4x*Grk* ^−/−^ neutrophils were differentially dye-labeled before they migrated side-by-side in the under-agarose gel system from a responder hole toward a source of chemoattractants (**Figure 1B**). Confirming our previous results in combined gradients of LTB4 and CXCL2, 4x*Grk* ^−/−^ neutrophils migrated within 4 hours two to three times further than WT cells in response to single gradients of the intermediary attractants LTB4 or CXCL2 (**Figures 1C, D**). Neutrophils can respond to immune cell-derived “intermediate-target” chemotactic factors, but they can also move in response to so called “end-target” chemoattractants, which are factors released from damaged cells or pathogens (38). We then tested migration toward end-target chemoattractants (1), which are not involved in swarm-amplification but rather in the initial recruitment to sites of damage or infection (**Figure 1E**). In contrast to *Grk2* ^−/−^ neutrophils (7), we observed significantly increased migration of 4x*Grk* ^−/−^ neutrophils towards W-peptides (ligands for the FPR1 and FPR2 receptors) and C5a (**Figures 1F, G**).

Thus, GRKs other than GRK2 contribute to the regulation of the chemotactic response toward end-target chemoattractants. Together, our data show that the complete depletion of all GRKs in neutrophils leads to an increase in GPCR-controlled migration across several GPCR types, supporting the canonical role of GRKs in regulating GPCR desensitization.

### T cells lacking GRK2 and GRK6 show distinct effects on S1PR-, CCR7- and CXCR4- controlled migration

**Figure 2:**
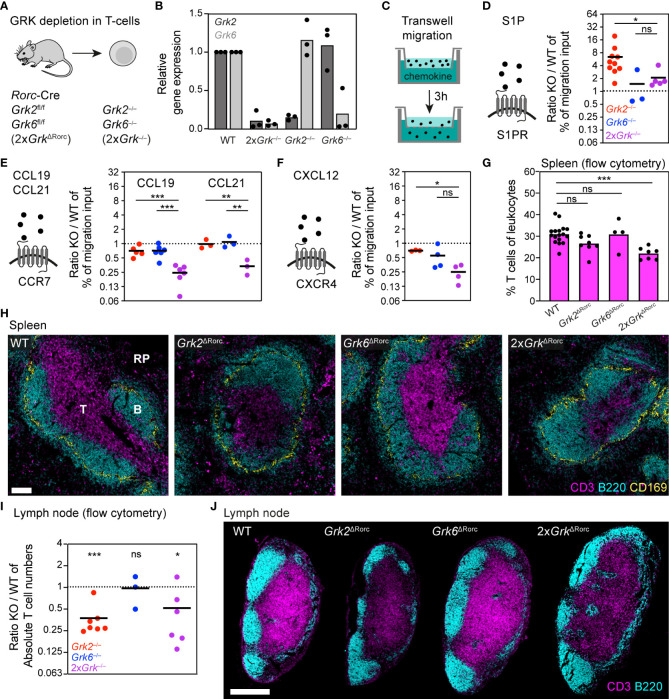
T cells lacking GRK2 and GRK6 show distinct effects on S1PR-, CCR7- and CXCR4- controlled migration **(A)** Scheme of the mouse model used for the generation of T cells with individual and combinatorial GRK depletion. **(B)** Confirmation of GRK knockout efficiency by quantitative RT-PCR. Relative gene expression of splenic *Grk*-deficient T cells, normalized to WT T cells. *n* = 3 biological replicates performed as independent experiments with *N*=3 technical replicates each. Bars display the mean. **(C)** Scheme of the transwell migration assay, in which cells migrate over 3 h from an upper compartment into an attractant-filled lower well. **(D–F)** Schemes of chemoattractants and their corresponding GPCRs. Transwell migration of splenic T cells toward 100 nM S1P **(D)**, 1 µg/ml CCL19 and 1 µg/ml CCL21 **(E)** and 0.5 µg/ml CXCL12 **(F)**. Displayed are ratios of *Grk*-knockout (KO) versus WT. *n* = 3-8 biological replicates performed as independent experiments with *N* = 2 technical replicates each. Bars display the mean. ***P < 0.001, **P < 0.01, *P < 0.05, ns nonsignificant, *post hoc* after or ANOVA. **(G)** Frequency of T cells in spleens compared to all leukocytes, measured by flow cytometry. *n* = 4-8 mice per gene knockout. Bars display the mean. **P<0.01, ns nonsignificant, *post hoc* after ANOVA. **(H)** Positioning of WT and *Grk*-deficient T cells in homeostatic spleens. Red pulp (RP), B cell follicles **(B)** and T cell zone (T) are indicated. T cells were detected with immunostaining against CD3 (magenta). B cells (B220, cyan) and marginal zone macrophages (CD169, yellow) are displayed for orientation. Scale bar: 100 µm. **(I)** Absolute numbers of T cells in inguinal lymph nodes measured by flow cytometry, displayed are ratios of KO vs. WT. *n* = 3-7 mice per KO. Bars display the mean. ***P < 0.001, *P<0.05, ns nonsignificant, one sample *t* test. **(J)** Positioning of WT and *Grk*-deficient T cells in homeostatic lymph nodes. T cells were detected with immunostaining against CD3. B cells (B220) are displayed for orientation. Scale bar: 300 µm.

As we aimed for a comparative analysis of GRK functions in different primary immune cells, we expanded our experiments to subsets of lymphocytes. We started with T cells, key immune cells of the adaptive immune response, which as naïve T cells constitutively recirculate between blood and lymphoid organs under homeostatic conditions (39). T cells mainly express two GRK family members, GRK2 and GRK6 (4, 5), which we simultaneously targeted by generating *Rorc-Cre Grk2^fl/fl^ Grk6^fl/fl^* (2x*Grk* ^ΔRorc^) mice (**Figure 2A**). This approach allowed us to isolate primary splenic T cells lacking both GRKs (2x*Grk* ^−/−^) and to analyze their chemotactic migration *in vitro* and homeostatic localization in lymphoid organs. Moreover, we could directly compare migratory responses of 2x*Grk* ^−/−^ T cells with single gene deficient *Grk2* ^−/−^ and *Grk6* ^−/−^ T cells (**Figure 2B**). By using transwell chemotaxis assays (**Figure 2C**), we systematically assessed the migration toward the most prominent chemoattractants and chemokines that promote the migration of naïve T-cells: sphingosin-1-phosphate (S1P), CCL19, CCL21 and CXCL12 (1).

Supporting the role of GRK2 as an important regulator of S1P-receptor (S1PR) desensitization (6), we confirmed previous findings that *Grk2* ^−/−^ T cells show a substantially increased migration response to varying concentrations of S1P (**Figure 2D**; **Supplementary Figures 1A, B**). In contrast, S1P-controlled migration of *Grk6* ^−/−^ T cells was normal (**Figure 2D**; **Supplementary Figures 1A, B**). Based on our findings with neutrophils, we assumed that in a situation of combined GRK deficiency, the phenotype of *Grk2* ^−/−^ T cells would still be reflected in T cells lacking both GRK2 and GRK6. However, we found that 2x*Grk* ^−/−^ T cells did not show the same substantial increase in chemotaxis as observed in cells lacking only GRK2. Instead, the additional loss of GRK6 in 2x*Grk* ^−/−^ T cells mitigated the lack of desensitization seen in *Grk2* ^−/−^ T cells (**Figure 2D**; **Supplementary Figure 1B**), which contrasts the comparison of *Grk2* ^−/−^ versus 4x*Grk* ^−/−^ neutrophils. Next, we tested the chemotactic behavior of T cells in response to the CCR7 ligands CCL19 and CCL21, homeostatic chemokines with important roles for T cell trafficking in lymphoid organs. We found that a deficiency of either GRK2 or GRK6 alone had only a weak (CCL19) or no (CCL21) influence on the migratory response of T cells. Surprisingly, however, a combined loss of GRK2 and GRK6 strongly inhibited T cell chemotaxis toward these chemokines (**Figure 2E**; **Supplementary Figures 1A, B**). Interestingly, we observed a similar pattern of GRK-mediated regulation, when T cells migrated towards the CXCR4 ligand CXCL12 (**Figure 2F**; **Supplementary Figures 1A, B**). Thus, combined depletion of GRK2 and GRK6 in T cells leads to migration deficiencies that are not detected upon depletion of an individual GRK. Moreover, the combined loss of both GRKs has differential effects for S1PR versus CCR7 and CXCR4 function, leading either to increased or reduced chemotactic migration, respectively.

As our *in vitro* experiments revealed contradicting roles for GRK-mediated T cell migration depending on the specific GPCR type, we were unable to predict how the combined loss of GRK2 and GRK6 would influence physiological T cell trafficking. Therefore, we analyzed T cell numbers and localization patterns in homeostatic spleens of mice. Confirming previous data from *Grk2* ^ΔCD4^ mice (6), we could not detect altered T cell numbers in *Grk2* ^ΔRorc^ mice in the spleen (**Figure 2G**). As a consequence of impaired S1PR-desensitization, *Grk2*-deficient T cells populate only sparsely the CCL21-rich T-cell zone, but accumulate in the blood- and S1P-rich red pulp (**Figure 2H**). In agreement with only weak phenotypes of *Grk6* ^−/−^ T cells *in vitro*, splenic T cell numbers and localization were unaffected in *Grk6* ^ΔRorc^ mice (**Figures 2G, H**). Importantly, the analysis of 2x*Grk2* ^ΔRorc^ mice revealed an overall reduction of splenic T cells numbers (**Figure 2G**), which was reflected in substantially reduced cell numbers in T-cell zones and red pulp (**Figure 2H**). Based on our *in vitro* characterization of 2x*Grk* ^−/−^ T cells, we argue that impaired attraction to CCR7 ligands is a major underlying cause for this T cell trafficking phenotype in the spleen. Next, we investigated the consequences of GRK depletions and misbalanced GPCR signaling for T cell homeostasis in lymph nodes (LNs). T cell numbers in LNs of *Grk2* ^ΔRorc^ mice and 2x*Grk2* ^ΔRorc^ mice were reduced by half (**Figure 2I**) with corresponding reductions of T cells in T-cell zones (**Figure 2J**). In contrast, T cell populations remained unaltered in *Grk6* ^ΔRorc^ mice (**Figures 2I, J**).

Together, our results show that the combined functions of GRK2 and GRK6 play an important role for T cells in balancing the response toward competing chemokines and localization signals. Individual loss of GRK2 leads primarily to an increase in S1PR-mediated chemotaxis. In contrast, the additional loss of GRK6 strongly decreases CCR7-controlled T cell migration. Both conditions, however, result in a defective positioning of T cells to their dedicated compartments in lymphoid organs.

### Combined depletion of GRK2 and GRK6 in B cells has opposite effects to those in T cells

**Figure 3:**
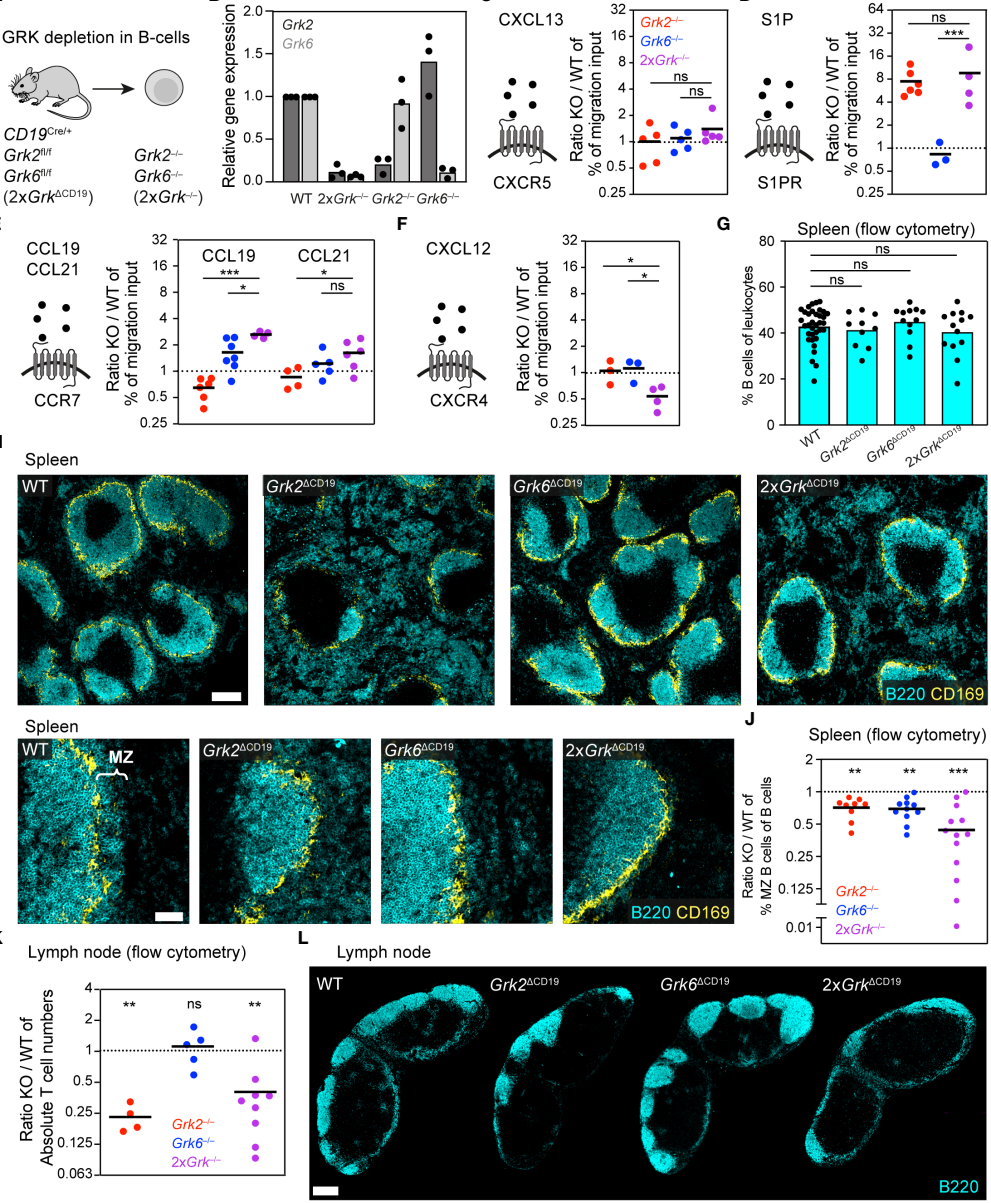
Combined depletion of GRK2 and GRK6 in B cells has opposite effects to those in T cells. **(A)** Scheme of the mouse model used for the generation of B cells with individual and combinatorial GRK depletion. **(B)** Confirmation of knockout efficiency by quantitative RT-PCR. Relative gene expression of splenic *Grk*-deficient B cells, normalized to WT. *n* = 3 biological replicates performed as independent experiments with *N* = 3 technical replicates each. Bars display the mean. **(C-F)** Schemes of chemoattractants and their corresponding GPCRs. Transwell migration of splenic B cells toward 0.5 µg/ml CXCL13 **(C)**, 100 nM S1P **(D)**, 1 µg/ml CCL19 and 0.1 µg/ml CCL21 **(E)**, 0.5 µg/ml CXCL12 **(F)**. Displayed are ratios of *Grk*-knockout (KO) versus WT. *n* = 3-7 biological replicates performed as independent experiments with *N* = 2 technical replicates each. Bars display the mean. ***P<0.001, **P<0.01, *P<0.05, ns nonsignificant, *post hoc* after ANOVA. **(G)** Frequency of B cells in spleens compared to all leukocytes, measured by flow cytometry. *n* = 10-13 mice per KO. Bars display the mean. ns nonsignificant, *post hoc* after ANOVA. **(H)** Positioning of WT and *Grk*-deficient B cells in homeostatic spleens. B cells were detected with immunostaining against B220 (cyan). Marginal zone macrophages (CD169, yellow) are displayed for orientation. Scale bar: 200 µm. **(I)** Zoom-in images of the splenic sections displayed in **(H)**, focusing on the marginal zone (MZ). Scale bar: 50 µm. **(J)** Frequency of splenic marginal zone B cells compared to all B cells, measured by flow cytometry. Displayed are ratios of KO versus WT. *n* = 9-13 mice per genotype. Bars display the mean. ***P<0.001, **P<0.01, one sample *t* test. **(K)** Absolute numbers of B cells in inguinal lymph nodes measured by flow cytometry, displayed are ratios of KO vs. WT. *n* = 4-9 mice per KO. Bars display the mean. **P<0.01, ns nonsignificant, one sample *t* test. **(L)** Positioning of WT and *Grk*-deficient B cells in homeostatic lymph nodes. B cells were detected with immunostaining against B220 (cyan). Scale bar: 300 µm.

B lymphocytes, key immune cells of the humoral component of the adaptive immune response, share many GPCRs with T lymphocytes, and we next addressed whether the regulation by GRKs differs between those cell types. Similar to T cells, the homeostatic trafficking of naïve B depends on the expression of S1PR, CCR7 and CXCR4. In addition, B cells express CXCR5 to sense CXCL13, the main B-cell follicle chemokine (40). Similar to T cells, B cells also express GRK2 and GRK6 as the major two GRK family members (4, 5). To simultaneously target them in B cells, we generated *CD19^CRE^ Grk2^fl/fl^ Grk6^fl/fl^* (2x*Grk* ^ΔCD19^) mice (**Figure 3A**). This approach allowed us to isolate splenic primary B cells lacking both GRKs (2x*Grk* ^−/−^), compare them to single gene deficient *Grk2* ^−/−^ and *Grk6* ^−/−^ B cells and proceed similar to our T cell analysis (**Figure 3B**).

When we analyzed the movement of B cells toward CXCL13, the CXCR5-dependent migration response was not altered by the absence of GRK2 and GRK6 (**Figure 3C**; **Supplementary Figures 1C, D**). Next, we studied B cell migration toward S1P (**Figure 3D**; **Supplementary Figures 1C, D**). We confirmed that *Grk2* ^−/−^ B cells showed strongly increased chemotaxis, supporting previous reports of GRK2-mediated S1PR-desensitization (6, 13). In contrast, *Grk6*-deficiency did not alter the chemotactic response to S1P. Thus, *Grk2* ^−/−^ and *Grk6* ^−/−^ B cells showed comparable phenotypes to T cells with the same single GRK deficiency. However, 2x*Grk* ^−/−^ T cells and 2x*Grk* ^−/−^ B cells had distinct phenotypes (**Figures 2D**, **3D**). In contrast to T cells, the additional loss of GRK6 in 2x*Grk* ^−/−^ B cells did not mitigate the pro-migratory effect of *Grk2* ^−/−^ B cells (**Figure 3D**; **Supplementary Figures 1C, D**). Further experiments investigated the chemotactic behavior of CCR7-expressing B cells to CCL19 and CCL21 (**Figure 3E**; **Supplementary Figures 1C, D**). Analogous to T cells, single *Grk2*-deficiency reduced B cell chemotaxis to CCL19 (6, 13). Unexpectedly, *Grk6* ^−/−^ B cells showed however an increase in chemotaxis, which was further pronounced in 2x*Grk* ^−/−^ B cells. Migration to CCL21 showed the same trends as to CCL19, albeit the migration phenotypes were less pronounced (**Figure 3E**; **Supplementary Figure 1C**). Together, we demonstrate that the combined depletion of GRK2 and GRK6 has opposite effects for the CCR7-controlled migration of T cells and B cells. While T cell movement deteriorates, the migration of B cells improves. Lastly, we also assessed the CXCR4-dependent B cell response toward CXCL12 and show that combined GRK2 and GRK6 depletion leads to impaired B cell migration (**Figure 3F**; **Supplementary Figures 1C, D**), similar to T cells (**Figure 2F**). Together, these experiments further exemplify that complete GRK deficiency causes entirely different migratory outcomes, depending on the specific immune cell subset and GPCR type.

Physiological B cell trafficking in secondary lymphoid organs depends on the sensing and interpretation of multiple chemoattractant and chemokine gradients. As our *in vitro* analysis revealed GRK mutant B cells with differential responsiveness for homeostatic attractants, we wondered whether and how this influences B cell localization in spleen and LNs. When we performed flow cytometric analysis of the percentages of splenic B cells (**Figure 3G**) or follicular B cells (**Supplementary Figure 1E**) in various B cell-specific GRK mutant mice, we did not detect gross differences between genotypes. Supporting previous work with *Mb1^Cre^ Grk2^fl/fl^* mice (6, 13), we confirmed that *Grk2* ^ΔCD19^ mice have substantially altered intra-splenic B cell distribution (**Figure 3H**). *Grk2* ^−/−^ B cells are drawn out of splenic B cell follicles into the red pulp. In contrast, spleens of *Grk6* ^ΔCD19^ showed no obvious changes in the localization of follicular B cells (**Figure 3H**). Strikingly, the additional lack of GRK6 in 2x*Grk* ^ΔCD19^ mice led to a partial rescue of splenic B cell distribution in comparison to *Grk2* ^ΔCD19^ mice. B cell follicle sizes were almost restored, and B cell accumulation in the red pulp was not as pronounced as in *Grk2* ^ΔCD19^ mice (**Figure 3H**, 4^th^ image). Thus, these observations suggest that the lack of S1PR1 desensitization, which is equally seen in *Grk2* ^−/−^ and 2x*Grk* ^−/−^ B-cells and drives *Grk2* ^−/−^ B-cells into the red pulp, is partially counter-balanced in 2x*Grk* ^−/−^ B cells by their altered responsiveness to CCR7 and CXCR4 ligands.

Besides follicular B cells, we also analyzed the population of marginal zone (MZ) B cells. Both immunofluorescence stainings (**Figure 3I**) and flow cytometric measurements (**Figure 3J**) revealed significantly reduced numbers of MZ B cells in *Grk2* ^ΔCD19^, *Grk6* ^ΔCD19^ and 2x*Grk* ^ΔCD19^ mice. These phenotypes could not be explained by changed integrin receptor expression or integrin-mediated adhesion, which remained unaltered (**Supplementary Figures 1F, G**).

Lastly, we also examined B cell numbers and distribution in LNs of mice. Similar to T cells in the LN, we measured strongly reduced B cell numbers (**Figure 3K**) and diminished sizes of B cell follicles (**Figure 3L**) in *Grk2* ^ΔCD19^ and 2x*Grk* ^ΔCD19^ mice. In addition, we observed a lack of B cells distributed around high-endothelial venules (HEVs) that are located within the T cell area (**Supplementary Figure 1H**).

Overall, our results demonstrate that the GRK-mediated regulation of GPCR function cannot be generalized across immune cell subsets. Although T and B lymphocytes share many general characteristics, including the expression of the major GRK family members (GRK2, GRK6) and the same cell surface GPCRs (S1PR, CCR7, CXCR4), we here show that the GRK-mediated control of GPCRs can be remarkably different between both cell types. In particular, our experiments with complete GRK depletions revealed some of these unexpected cell-type specific roles of GRKs and the need to consider the specific GPCR type in a defined immune cell subset.

### Combined GRK depletion in dendritic cells impairs their migratory properties

We completed our systematic analysis of GRK-mediated regulation of immune cell migration by studying dendritic cells (DCs), important immune cells with a bridging role between the innate and the adaptive immune systems. These cells reside in peripheral and lymphoid tissues where they acquire antigens, which they process and then present to T cells (41). Although it is known that DCs express three members of the GRK family (GRK2, GRK3 and GRK6), we have only limited insight into their function for DC biology. While single gene knockouts of *Grk3* or *Grk6* were previously used to study BMDC migration *in vitro* (9, 15), we are completely missing any knowledge on the functional role of GRKs for DC homeostasis *in vivo*.

**Figure 4:**
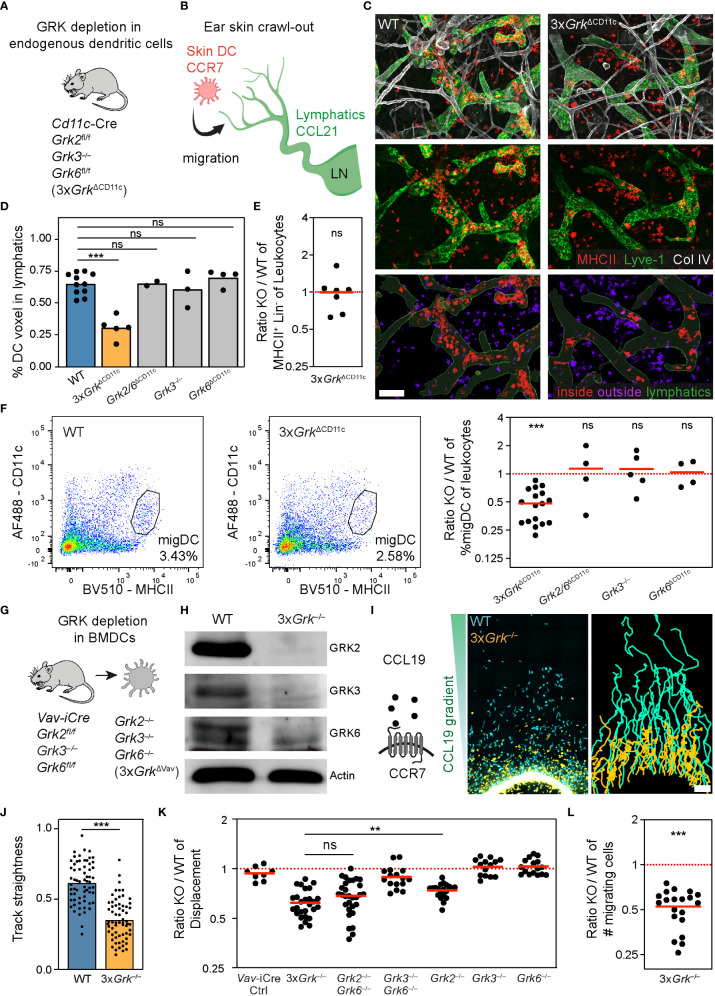
Complete GRK depletion in dendritic cells impairs their migratory properties. **(A)** Scheme of the mouse model used to analyze endogenous DCs with individual and combinatorial GRK depletion. **(B)** Scheme of the ear skin crawl-out assay. Endogenous skin-resident DCs migrate from the skin into dermal lymphatics. DCs express the chemokine receptor CCR7 and sense the chemokine CCL21 expressed by lymphatic endothelial cells. **(C)** *Ex vivo* migration of skin WT and *Grk*-deficient DCs into lymphatic vessels (crawl-out assay, 16 h after start). Upper and middle rows: DCs are detected by immunostaining against MHCII (red) and lymphatics by anti-Lyve-1 staining (green). Collagen IV (white) shows basement membranes of vessels, nerves and fat cells, and is displayed for orientation. Images are projections of several confocal *z*-planes. Lower row: image analysis to quantify DCs inside (red) and outside (purple) of lymphatic vessels (green). Scale bar: 100 µm. **(D)** Quantification of endogenous DC migration (crawl-out assay). *n* = 2-5 mice per gene knockout with *N* = 5-12 different imaging fields of view per mouse. Bars display the mean. ***P < 0.001, ns nonsignificant, *post hoc* after Kruskal-Wallis test. **(E)** Number of skin DCs compared to all skin leukocytes was determined by flow cytometry. Displayed is the ratio of 3x*Grk* ^ΔCD11c^ (KO) versus WT. *n* = 7 mice per genotype. Bar displays the mean. ns nonsignificant, one sample *t* test. **(F)** Representative flow cytometric analysis of migratory DCs (migDC) in inguinal lymph nodes (left). Quantification displayed as ratio of KO versus WT (right). *n* = 4-17 mice per genotype. Bars display the mean. ***P < 0.001, ns nonsignificant, one sample *t* test. **(G)** Scheme of the mouse model used for the generation of BMDCs with individual and combinatorial GRK depletion. **(H)** Confirmation of the knockout efficiency by immunoblot analysis. GRK2, GRK3 and GRK6 protein expression levels in lysates of WT and 3x*Grk* ^–/–^ BMDCs. Actin was used as loading control. **(I)** Scheme of the chemoattractant CCL19 and the corresponding chemokine receptor CCR7. Migration of WT and 3x*Grk* ^–/–^ BMDCs in under-agarose assay toward CCL19 was recorded with live-cell microscopy. Cell displacement after 12 h (left) and cell tracks over 12 h (right) is shown. Scale bar: 150 µm. **(J)** Quantification of DC mean track straightness from *n* = *3* biological replicates performed as independent experiments with *N* = 15-25 tracked cells per experiment. Bars display the mean. ***P < 0.001, *t* test. **(K)** Endpoint analysis of mean displacement ratios (KO versus WT) after 16 h. *n* = 1 (Vav-iCre Ctrl), *n*=2 (*Grk3* ^–/–^ *Grk6* ^–/–^), *n* = 4 (*Grk2* ^–/–^ *Grk6* ^–/–^) or *n* = 3 (all others) biological replicates with *N* = 3-8 technical repeats per experiment. Bars display the mean. **P < 0.01, ns nonsignificant, *post hoc* after ANOVA. **(L)** Endpoint analysis of number of cells that migrated under the agarose given equal input numbers. Displayed is the ratio 3x*Grk* ^–/–^ versus WT cells. *n*=3 biological replicates performed as independent experiments with *N* = 3-8 technical repeats per experiment. Bar displays the mean. ***P < 0.001, one sample *t* test.

This prompted us to simultaneously target all three GRKs by generating *CD11c-Cre Grk2^fl/fl^ Grk3* ^−/−^ *Grk6^fl/fl^* (3x*Grk* ^ΔCD11c^) mice (**Figure 4A**), which allowed the study of endogenous DCs depleted of all expressed GRKs. In contrast to neutrophils and lymphocytes, which respond to many different chemoattractants and chemokines, the migration of activated DCs is predominantly driven by CCR7 ligands. CCR7-expressing DCs sense gradients of CCL21 at lymphatic vessels and in LNs, which thus determine the migratory route of peripheral DCs from the tissue periphery into the secondary lymphoid organ (**Figure 4B**). The first step of this migratory route, leaving the interstitium and entering skin lymphatic vessels, can be visualized in the ear skin crawl-out assay (**Figure 4B**; **Supplementary Figure 2A**). In this experimental setup, the *ex vivo* culture of ear skin halves over 16 h activates endogenous skin-resident DCs and triggers their migration into lymphatics (**Figure 4C**; **Supplementary Figure 2A**). We quantified immunofluorescence stainings of DCs inside and outside of lymphatics in tissue regions of comparable lymphatic vessel density (**Supplementary Figure 2B**) and found that the majority of WT DCs migrated into the lymphatics during this timeframe (**Figures 4C, D**). In striking contrast, only a small fraction of endogenous skin DCs entered lymphatic vessels in the ears of 3x*Grk* ^ΔCD11c^ mice (**Figure 4D**), although flow cytometric analysis measured comparable numbers of CD11c^+^ MHCII^+^ cells between these knockout and control mice (**Figure 4E**). This phenotype was only observed in ears of triple GRK knockout mice and could not be detected in ears of mice with single or double GRK knockout combinations (**Figure 4D**). After entering the lymphatics, DCs follow them until they reach the draining LNs. This migration also takes place under homeostasis and the number of endogenous migratory DCs in skin-draining LNs can be assessed based on their very high MHCII-expression levels in comparison to LN-resident DCs (**Figure 4F**). In agreement with our results from the ear skin crawl-out assay, we measured reduced fractions of migratory DCs in the skin-draining LNs of 3x*Grk* ^ΔCD11c^ mice. This phenotype was again specific to triple GRK knockout mice and could not be measured in mice deficient for either GRK3 or GRK6, or a combination of GRK2 and GRK6 (**Figure 4F**).

To study DC migration in more detail, we generated bone marrow-derived DCs (BMDCs) from bone marrow precursor cells in GM-CSF culture medium. We used bone marrow from either 3x*Grk* ^ΔCD11c^ mice or *Vav-iCre Grk2* ^fl/fl^ *Grk3* ^−/−^ *Grk6* ^fl/fl^ (3x*Grk* ^ΔVav^) mice, which have early Cre activity in hematopoietic cell precursor stages, and found more efficient GRK depletion in BMDCs from 3x*Grk* ^ΔVav^ mice (data not shown). GRK depletion in BMDCs from 3x*Grk* ^ΔVav^ mice resulted in an almost complete loss of all expressed GRKs (3x*Grk* ^−/−^) (**Figures 4G, H**). Lipopolysaccharide (LPS) stimulation for 24h causes BMDCs to acquire a mature phenotype and develop into DCs that chemotactically respond to CCR7 ligands. Side-by-side comparison of LPS-matured 3x*Grk* ^−/−^ BMDCs with control cells in under-agarose chemotaxis assays revealed a pronounced chemotaxis defect of the triple GRK knockout DCs in response to CCL19 gradients (**Figure 4I**; **Supplementary Video 1**). The migration track straightness of 3x*Grk* ^−/−^ DCs was severely reduced (**Figure 4J**), which resulted in a drop of total displacement towards the chemokine source (**Figure 4K**). More detailed analysis revealed that this defect is present during the whole migration time, but more pronounced in the late phase (200-700 min) (**Supplementary Figures 2C, D**). The migration speeds of control and 3x*Grk* ^−/−^ BMDCs were comparable for both phases (**Supplementary Figure 2E**). Not only did we observe that 3x*Grk* ^−/−^ DCs show reduced total displacement, but also that less cells responded to the stimulus and migrated out of the responder hole (**Figure 4L**). We also assessed the migration of BMDCs with single or double *Grk*-deficiency. Confirming our *in vivo* findings, *Grk3* ^−/−^ and *Grk6* ^−/−^ BMDCs displaced normally toward CCL19 gradients (**Figure 4K**). A migration-dampening effect in this *in vitro* setup was observed for *Grk2* ^−/−^ and *Grk2/6* ^−/−^ BMDCs (**Figure 4K**). As we had however not observed phenotypes of these KO combinations *in situ* and *in vivo* (**Figures 4D, F**), we decided to focus on the analysis of 3x*Grk* ^−/−^ DCs for all following experiments.

Together, our experiments with single and combinatorial GRK depletions in DCs do not support a role for this kinase family in controlling CCR7 desensitization during chemotactic migration. Instead, we find that complete GRK depletion in 3x*Grk* ^−/−^ DCs results in defective CCR7-mediated migration *in situ* and *in vivo*.

### Complete GRK depletion impairs DC development *in vitro*

**Figure 5:**
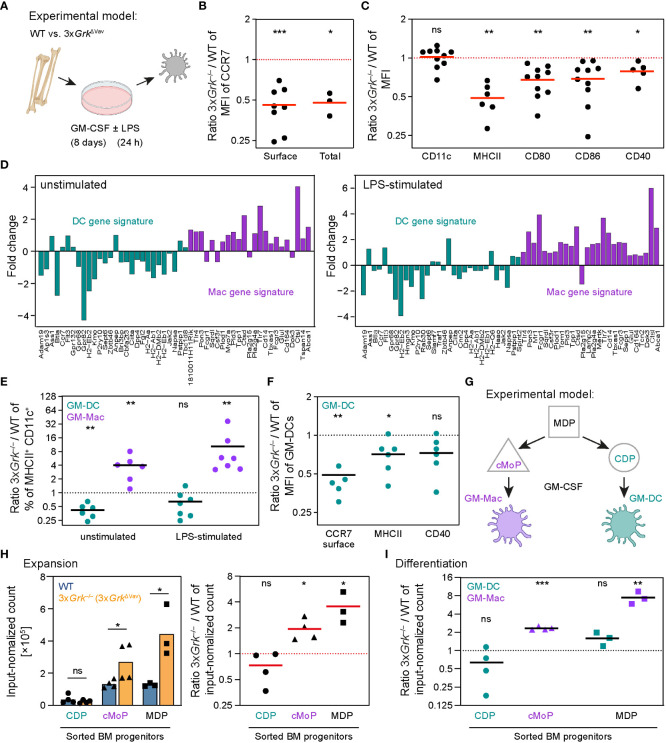
Complete GRK depletion impairs DC development *in vitro*. **(A)** Scheme of the generation of BMDCs in GM-CSF culture. After 8 days of culture, cells were either left untreated (unstimulated) or stimulated with LPS (LPS-stimulated) for 24 h. **(B, C)** Quantification of cell surface and total CCR7 expression levels **(B)** and surface expression of other markers **(C)** in CD11c^+^ LPS-stimulated BMDCs. Displayed are ratios of 3x*Grk* ^–/–^ versus WT. *n* = 3-8 biological replicates performed as independent experiments. Bars display the mean. **P<0.01, *P<0.05, one sample *t* test. **(D)** Bulk RNA-Sequencing of unstimulated (left) and LPS-treated (right) WT and 3x*Grk* ^–/–^. WT and knockout cells were compared. Fold changes of differentially expressed genes belonging to either a DC (petrol) or macrophage (Mac, purple) gene signature are displayed. **(E)** Quantification of bona fide DCs (GM-DC) and macrophages (GM-Mac) developing in GM-CSF cultures. Displayed are ratios 3x*Grk* ^–/–^ versus WT with or without LPS stimulation. *n* = 6-7 biological replicates performed as independent experiments. Bars display the mean. **P<0.01, ns nonsignificant, one sample *t* test. **(F)** Quantification of surface expression levels of DC markers in LPS-stimulated GM-DCs. Displayed are ratios 3x*Grk* ^–/–^ versus WT with or without LPS stimulation. *n* = 6 biological replicates performed as independent experiments. Bars display the mean. **P<0.01, *P<0.05, ns nonsignificant, one sample *t* test. **(G)** Scheme of precursors generating GM-DCs and GM-Macs in GM-CSF BM cultures. Macrophage-DC progenitors (MDP) can give rise to common DC precursors (CDPs) and common monocyte precursors (cMoPs). **(H)** Flow cytometric quantification of the proliferation rate of sorted BM progenitors (CDP, cMoP or MDP) in unstimulated GM-CSF cultures. Displayed are numbers normalized to the input (left) and ratios of 3x*Grk* ^–/–^ versus WT (right). *n* = 3-4 biological replicates performed as independent experiments. Bars display the mean. *P<0.05, ns nonsignificant, *t* test (left side CDP,MDP), Mann-Whitney test (left side cMoP), one sample *t* test (right side). **(I)** Progenitors (CDP, cMoP or MDP) were sorted from bone marrow before they were grown in GM-CSF-containing medium. After 8 days, differentiation into GM-Mac and GM-DC was analyzed by flow cytometry. *n* = 3-4 biological replicates performed as independent experiments. Bars display the mean. ***P<0.001, **P<0.01, ns nonsignificant, one sample *t* test.

The migration defect of LPS-matured 3x*Grk* ^−/−^ BMDCs prompted us to examine these GM-CSF cultured cells in more detail (**Figure 5A**). As CCR7 is critical for the migration of mature DCs, we analyzed its expression in LPS-matured BMDCs. Flow cytometric analysis showed that both CCR7 cell surface expression and CCR7 total expression, including the intracellular pool, were reduced in LPS-stimulated 3x*Grk* ^−/−^ BMDCs in comparison to WT cells (**Figure 5B**). To clarify whether this resulted from a generalized DC maturation defect, we measured other typical cell surface molecules of mature DCs. Indeed, several DC maturation markers, including MHCII, CD80, CD86 and CD40, were diminished on the cell surface of LPS-matured 3x*Grk* ^−/−^ BMDCs (**Figure 5C**). However, the cell surface expression levels of the classical DC marker CD11c were unaltered (**Figure 5C**). As these results suggested defective DC maturation, we sought to confirm it by performing bulk RNA sequencing on unstimulated and LPS-matured 3x*Grk* ^−/−^ and WT BMDCs. To our surprise, these analyses revealed that not only maturation-related, but also many more genes that define a bona fide DC gene signature (42) were downregulated in 3x*Grk* ^−/−^ BMDCs (**Figure 5D**, petrol). Instead, a macrophage (Mac) gene signature (43) emerged in both unstimulated and LPS-stimulated cells (**Figure 5D**, purple).

Previous work revealed that GM-CSF cultured BMDCs are a heterogeneous myeloid cell population consisting of bona fide DCs (termed “GM-DC”) and a minor fraction of macrophage-like CD11c^+^ MHCII^+^ cells (termed “GM-Mac”) (30). When we refined our flow cytometric analysis for distinguishing GM-DCs and GM-Macs in BMDC cultures, we found a clear shift between both cell populations in 3x*Grk* ^−/−^ BMDC cultures. Among CD11c^+^ MHCII^+^ cells, the fraction of GM-DCs was decreased, whereas GM-Macs were markedly increased in both unstimulated and LPS-stimulated 3x*Grk* ^−/−^ GM-CSF cultures (**Figure 5E**). Importantly, the remaining bona fide GM-DCs, which still developed in 3x*Grk* ^−/−^ cultures, also showed reduced expression of CCR7, MHCII and CD40 (**Figure 5F**). Together, these findings provide an explanation for the two observed phenotypes of 3x*Grk* ^−/−^ cells in the under-agarose chemotaxis assays; (a) less responding cells, because GM-Macs do not migrate to CCL19 (30), and (b) reduced displacement of the responding cells, because the remaining bona fide GM-DCs remain lowered in CCR7-expression. Additionally, we noticed that not only DCs derived from GM-CSF cultures showed an altered phenotype, but also DC development in the presence of Flt3 ligand was impaired. In particular, the number of CD11b^−^ DCs was strongly reduced, whereas the CD11b^+^ DC population was not significantly altered (**Supplementary Figure 3A**).

In the following experiments, we focused on a more detailed analysis of the development of GM-DCs and GM-Macs in 3x*Grk* ^−/−^ cultures. It has been shown that different bone marrow-derived precursors contribute to both cell states: GM-DCs derive from common DC precursors (CDPs), GM-Macs from common monocyte precursors (cMoPs). CDPs and cMoPs derive from macrophage-DC progenitors (MDPs) as common progenitor, which has the highest proliferation potential (30) (**Figure 5G**). Refined flow cytometric analysis of the bone marrow from 3x*Grk* ^ΔVav^ mice revealed decreased numbers of MDPs and CDPs, which suggests functional roles of GRKs for early hematopoietic stem cell stages (**Supplementary Figure 3B**). The very limited numbers of MDPs and CDPs in 3x*Grk* ^ΔVav^ mice made it impractical to sort these cells for further analyses. Instead, we used for the following experiments cells generated from 3x*Grk* ^ΔCD11c^ mice, whose bone marrow precursors do not yet express the Cre recombinase. Therefore, the frequencies of MDPs and CDPs are normal in these mice (**Supplementary Figure 3C**). Importantly, cultures from bone marrows of 3x*Grk* ^ΔCD11c^ mice showed the same, albeit mitigated, phenotypic hallmarks as cultures from bone marrow of 3x*Grk* ^ΔVav^ mice: defective BMDC displacement in under-agarose assays (**Supplementary Figure 3D**), a trend to reduced expression of DC maturation markers (**Supplementary Figure 3E**) and increase of GM-Macs over GM-DCs (**Supplementary Figure 3F**). To delineate whether the observed misbalance of GM-Macs/GM-DCs could be attributed to a specific differentiation path, we sorted CDP, cMoP and MDP cells from the bone marrow of WT or 3x*Grk* ^ΔCD11c^ mice and followed their expansion in GM-CSF culture medium. As expected, cMoPs and MDPs from WT mice were more proliferative than CDPs (**Figure 5H**). In comparison to WT cultures, the output of differentiated cells from CDPs of 3x*Grk* ^ΔCD11c^ mice was slightly decreased, whereas cMoPs and MDPs expanded more strongly (**Figure 5H**). Furthermore, MDPs did not differentiate into GM-DCs and GM-Macs at regular proportions. Instead, their increased proliferation generated mainly GM-Macs (**Figure 5I**).

Taken together, these results show that GRKs play a role in BMDC development with subsequent consequences for the migration response. Complete GRK depletion leads to a vastly increased number of CD11c^+^ macrophages in GM-CSF cultures, and those DCs, that still develop, are phenotypically impaired.

### Complete GRK depletion impairs DC differentiation *in vivo*

Having identified that complete GRK depletion shifts the development of GM-DCs toward GM-Macs *in vitro*, we next asked whether the development of endogenous DCs might also be altered. In particular, we were interested in the DC populations of secondary lymphoid organs, where several DC subsets can be distinguished. Two main populations of classical DCs (cDC1 and cDC2), which differ in origin and function, have previously been defined. Moreover, plasmacytoid DCs (pDCs) represent another DC subset in lymphoid organs.

**Figure 6:**
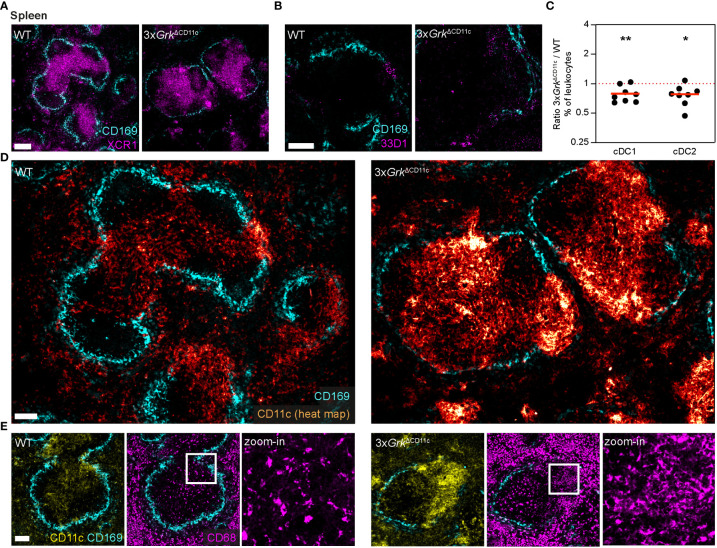
Complete GRK depletion impairs DC differentiation in the spleen. **(A, B)** Positioning of DC subsets in the spleens of 3x*Grk* ^ΔCD11c^ and littermate control mice. cDC1 were detected with immunostaining against XCR1 (A, magenta) and cDC2 with anti-33D1 (B, magenta). Marginal zone macrophages (CD169, cyan) are displayed for orientation. Scale bars: 200 µm **(A)**, 100 µm **(B)**. **(C)** Quantification of splenic DC subsets compared to all leukocytes by flow cytometry. Displayed are ratios of 3x*Grk* ^ΔCD11c^ versus WT. *n* = 8 mice per genotype. Bars display the mean. **P < 0.01, *P < 0.05, one sample *t* test. **(D)** Representative images of CD11c-expressing cells (glow heat map displays signal intensity) in spleens of 3x*Grk* ^ΔCD11c^ and littermate control mice. Marginal zone macrophages (CD169, cyan) are displayed for orientation. Scale bar: 100 µm. **(E)** Expression of the macrophage marker CD68 (magenta) in spleens of 3x*Grk* ^ΔCD11c^ (right) and littermate control mice (left). Zoom-in shows CD68 staining in spleen areas of high CD11c (yellow) signal. Marginal zone macrophages (CD169, cyan) are displayed for orientation. Scale bar: 100 µm (left, middle), 30 µm (zoom-in).

First, we used immunofluorescence stainings to detect cDC1 and cDC2 in spleens, where these DC subsets have distinct localization patterns (44). Using the specific markers XCR1 (for cDC1) and 33D1 (for cDC2) we found comparable localization of both DC subsets in WT and 3x*Grk* ^ΔCD11c^ animals: cDC1s positioned in the T-cell zone of the white pulp (**Figure 6A**) and cDC2s in the splenic bridging channels (**Figure 6B**). Flow cytometric analysis revealed that the abundance of *3xGrk* ^−/−^ cDC1s and cDC2s in comparison to all other immune cells was slightly decreased (**Figure 6C**), but the overall homeostasis of tissue-resident DCs appeared only mildly disturbed in 3x*Grk* ^ΔCD11c^ mice. Immunofluorescence stainings against CD11c revealed however a striking and unexpected phenotype. Massive accumulations of CD11c^+^ cells, mainly localized in the T-cell zones of the white pulp and bridging channels, were visible in the spleens of 3x*Grk* ^ΔCD11c^ mice (**Figure 6D**). Similar to cDCs, also pDCs could not account for this observation, as pDC numbers were comparable in spleens of WT and 3x*Grk* ^ΔCD11c^ mice (**Supplementary Figure 4A**). As our *in vitro* results showed increased CD11c^+^ GM-Macs in *3xGrk* ^−/−^ GM-CSF cultures, we next tested several macrophage and monocyte markers to characterize the accumulating CD11c^+^ population *in vivo*. CD11c^+^ cells in the T cell cortex did not co-express CD169, CD115 or F4/80 (**Figure 6D**; **Supplementary Figures 4B, C**). Next, we analyzed the expression of CD68, which is often used as macrophage-marker, but can also be expressed by other cells of myeloid origin (45). While in WT splenic white pulp only discrete, very bright signals were visible, which correspond to cortical macrophages, we detected an additional diffuse CD68 staining in the bridging channels of 3x*Grk* ^ΔCD11c^ spleens (**Figure 6E**), suggesting a macrophage-like phenotype of the accumulating CD11c^+^ cells.

**Figure 7:**
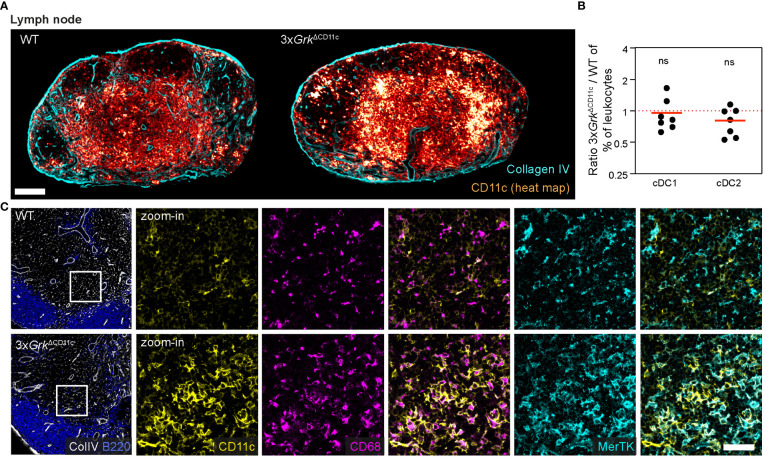
Complete GRK depletion impairs DC differentiation in the lymph node. **(A)** Representative images of CD11c-expressing cells (glow heat map displays signal intensity) in inguinal lymph nodes of 3x*Grk* ^ΔCD11c^ and littermate control mice. Collagen IV (cyan) is displayed for orientation. Scale bar: 200 µm. **(B)** Quantification of lymph node DC subsets compared to all leukocytes by flow cytometry. Displayed are ratios 3x*Grk* ^ΔCD11c^ versus WT. *n* = 7 mice per genotype. Bars display the mean. ns nonsignificant, one sample *t* test. **(C)** CD68 (magenta) and MerTK (cyan) expression in the T cell area of 3x*Grk* ^ΔCD11c^ and WT LNs in comparison to CD11c (yellow). B220 (blue) and colIagen IV (white) are displayed for orientation. Scale bar: 40 µm.

Lastly, we analyzed whether these macrophage-like CD11c^+^ cells were only present in spleens. Strikingly, we also detected an accumulation of CD11c^+^ cells in LNs of 3x*Grk* ^ΔCD11c^ mice, where these cells localized to the peripheral parts of the T-cell zone or distributed in smaller patches within the T-cell zone (**Figure 7A**). At the same time, the numbers of cDC1 and cDC2 in LNs were unchanged (**Figure 7B**) and the number of pDCs in LNs reduced in 3x*Grk* ^ΔCD11c^ mice (**Supplementary Figure 4A**). Analogous to the splenic population, also this LN population was CD68^+^ and additionally expressed MerTK (**Figure 7C**), a cell surface receptor expressed by many macrophage subsets.

In summary, 3x*Grk* ^ΔCD11c^ animals exhibit an accumulation of CD11c^+^ cells in secondary lymphoid organs, which did not resemble any conventional DC or macrophage population. Based on the expression of CD11c, CD68 and MerTK, we presume that these cells are of myeloid origin and phenotypically “in-between” macrophages and DCs. Presumably, they are the *in vivo* equivalent of the GM-Macs, which develop excessively in 3x*Grk* ^−/−^ GM-CSF cultures. Thus, complete GRK depletion in DCs has major consequences for DC development *in vitro* and *in vivo*.

## Discussion

We here studied systematically how combinatorial depletions of GRKs impact the GPCR-controlled migration of several primary mouse immune cells and their *in vivo* trafficking. Based on complex mouse genetics, our experiments allowed the characterization of immune cells that were missing major GRK-mediated kinase activities, including neutrophils lacking GRK2, GRK3, GRK5, GRK6, T cells and B cells lacking GRK2 and GRK6, and DCs lacking GRK2, GRK3 and GRK6. Previous studies have followed similar experimental strategies and knocked out several GRK isoforms in other cell types. HEK293 cell lines lacking GRK2, GRK3, GRK5 and GRK6 have recently been described and serve as valuable experimental tools for studying the impact of GRKs on GPCR-arrestin binding and GPCR regulation (46–48). However, there is only limited use of HEK293 cells for the study of GPCR-controlled migration responses. Combinatorial GRK depletions in primary cells have rarely been described. A previous study on fetal mouse heart development used mice with *Nkx2-5-Cre* mediated conditional ablations of GRK2, GRK5 and GRK6. Analysis of these animals revealed that these three GRKs redundantly modulate the 7-transmembrane spanning protein Smoothened and its crosstalk with the GATA transcriptional pathway, providing an example where only triple, but not double or single, GRK deficiency, results in a disease phenotype (49).

Our comprehensive comparison of mouse leukocyte subsets shows that combinatorial depletions of GRKs have pleiotropic and cell-type specific effects in primary immune cells *in vitro* and *in vivo*, many of which could not have been predicted. Neutrophils lacking all four major GRK family members show increased GPCR-controlled migration responses across several GPCR types, supporting the canonical role of GRKs in regulating GPCR desensitization (4, 7, 37). In contrast, combinatorial GRK depletion in T cells and B cells provides a more complex pattern of GPCR-controlled migration.

Previous studies on T and B cells with single GRK gene depletions served as basis for our experiments. When analyzing *Grk2/Grk6* double-deficient (2x*Grk* ^−/−^) lymphocytes, we performed control group experiments with *Grk2* ^−/−^ and *Grk6* ^−/−^ lymphocytes, which confirmed most previously published data. *Grk2* ^−/−^ T cells showed increased migration toward S1P, but lowered movement to CCR7 ligands and CXCL12 (6). *Grk6* ^−/−^ T cells were slightly impaired in their migration to CXCL12 (14). *Grk2* ^−/−^ B cells showed increased migration toward S1P, but lowered movement to CCR7 ligands (6, 13). *Grk6* ^−/−^ B cell migration was unaltered in response to CXCL12 (14). As shown before (6), *Grk2* ^−/−^ B cells did not show significantly impaired migration responses to CXCL13. However, this contrasted another study, maybe due to minor variations in the experimental setup (13). Our *in vivo* results from reduced T cell numbers in homeostatic spleens and LNs of *Rorc-Cre Grk2^fl/fl^* mice matched previous data in *Cd4-Cre Grk2^fl/fl^* mice (6). Our *in vivo* findings on severely altered B cell localization in spleens from *Cd19^Cre^ Grk2^fl/fl^* mice were comparable to previous studies in *Mb1^Cre^ Grk2^fl/fl^* mice (6, 13). As none of the previous work addressed the functional consequences of combinatorial GRK depletion in these cell types, we focused on this important aspect of GRK regulation.

First, we demonstrate for several GPCRs that the depletion of both GRK2 and GRK6 resulted in migration phenotypes that were not seen in single gene knockout controls. In T cells, *Grk2/Grk6* double-deficiency results in the inhibition of CCR7- and CXCR4-mediated migration and the mitigation of the increased S1PR-controlled movement seen in *Grk2* ^−/−^ cells. In B cells, combined loss of GRK2 and GRK6 leads to an increase in CCR7-mediated migration, but inhibition of CXCR4-induced migration and S1PR-controlled movement similar to *Grk2* ^−/−^ cells. Second, these results show that *Grk2/Grk6* double-deficiency yields similar (CXCR4), differential (S1PR) and opposing (CCR7) migration phenotypes between T and B cells. Together, these experiments exemplify how the combined depletion of two GRK isoforms results in distinct migratory outcomes for (a) different GPCRs in one immune cell type, and (b) one specific GPCR in different immune cell subsets. Furthermore, we show how the altered attractant responsiveness of *Grk2/Grk6* double-deficient lymphocytes influences their homeostatic positioning in secondary lymphoid organs, where T cells and B cells need to interpret complex gradients of chemokines and chemoattractants.

We here specifically focused on the analysis of GPCR-controlled cell migration, one of the major cellular processes downstream of GPCR signaling, which orchestrates the dynamics and organization of immune cells in lymphoid and non-lymphoid organs (1). We did not attempt to identify and dissect the detailed molecular mechanisms that underlie the observed migration phenotypes upon combinatorial GRK depletion. Given the important role of GRKs for phosphorylating agonist-occupied GPCRs at serine and threonine residues, it is very likely that the *Grk*-deficiency-induced migration phenotypes result from missing GRK functions directly at the GPCRs. GRK-dependent receptor phosphorylation can promote the recruitment and high affinity binding of arrestins, which uncouple the receptor from G protein, target receptors for internalization and promote G protein-independent signaling (50). Thus, we expect that the combinatorial depletion of highly expressed GRK family members in immune cells leads to substantial changes in GPCR phosphorylation, as basis for many altered cellular downstream processes. However, direct measurements of GRK-mediated phosphorylation at GPCR intracellular parts are very challenging with primary immune cell subsets. Most of our detailed knowledge on GRK-controlled GPCR phosphorylation derives from GPCR mutagenesis studies in easily transfectable cell lines (e.g. HEK293 cells), where candidate serine and threonine residues are substituted to alanines to prevent phosphorylation. Alternatively, cell lines are transfected with tagged GPCRs, which are then pulled down and applied to mass spectrometry (51). Direct antibody-based pull-downs of GPCRs do often not result in sufficient amounts of C-terminal peptides for mass spectrometry measurements. Given the well-known problems of insufficient cell material and inefficient gene delivery into primary immune cells, most of these methods are practically not feasible for studying GPCR phosphorylations in our cell types of interest. Phosphosite-specific antibodies are valuable tools for analyzing serine/threonine phosphorylations at specific GPCRs and can also be used with primary immune cells (51–53). However, such antibodies are difficult to produce and not yet available for every GPCR expressed in mammals. And even if available, phosphorylations through kinases other than GRKs, e.g. protein kinases A and C, also have to be considered (53). CXCR4 is probably the best-studied immune-related chemokine receptor in mouse leukocytes, as phosphosite-specific antibodies have been developed against its C-terminal tail (54, 55). Antibodies directed against specific phosphosites of CXCR4 have recently been used to delineate its sequential phosphorylation by GRK2/GRK3 and GRK6 in primary mouse B cells (19). Based on our comparative migration analysis of *Grk2* ^−/−^, *Grk6* ^−/−^ and *Grk2* ^−/−^ */Grk6* ^−/−^ T and B cells, we identified S1P receptors, most likely S1PR1, and CCR7 as the most interesting candidates for GRK-mediated GPCR regulation. The intracellular parts of S1PR1 and CCR7 have several serine and threonine residues, which can be subject to GRK-mediated phosphorylation (18, 56–59). Unfortunately, phosphosite-specific antibodies against these mouse GPCRs are not yet available to detect such phosphorylation-dependent changes in primary mouse immune cells. As we are missing such data, we cannot rule out compensation of the lack of individual GRK activity by remaining GRKs or other kinases.

Together, our experiments clearly show cell type-specific effects of combined GRK depletion for GPCR-controlled migration of immune cells. Differential phosphorylation patterns of a specific GPCR in different cell types might be an underlying mechanism of our findings. Several examples, mostly of non-immune cell related GPCRs, support the concept that GPCRs can be phosphorylated in a unique manner that is associated with the cell type in which the receptor is expressed (53). The pattern of GPCR hyperphosphorylation has been proposed to generate a “barcode” that can vary between cell types and influence the recruitment of arrestins (60–62). Binding of arrestins can interfere with GPCR signaling, but also generates a scaffold for more than 100 proteins that form specific effector hubs for controlling GPCR trafficking and intracellular signaling (50, 61). Thus, the same GPCR subtype can have different signaling properties depending on the cell type in which the receptor is expressed, which has been exemplified for the M_3_-muscarinic receptor, β_2_-adrenergic receptor and CXCR4 expressed in non-immune cells (54, 60, 63, 64). Although often still underappreciated, there is an increasing awareness that the composition and expression of GPCR-regulating proteins, including GRK family members, other protein kinases and arrestin isoforms, are different in each cell type, resulting in cell-type specific signaling outcomes (53, 61). Thus, comparisons between experiments with primary cells and complementary experimentation in cell lines need to be interpreted with caution, as the cell type-specific differences may not always allow meaningful conclusions.

Given the limitations of manipulating primary immune cells, we cannot fully rule out that the migration phenotypes, which we observed in double and triple-GRK knockout immune cells, are caused by missing GRK functions other than phosphorylation of active GPCRs (50). It has now been well established that GRKs can exert GPCR-independent signaling functions. First, GRKs can phosphorylate numerous non-GPCR substrates, including receptor tyrosine kinases, single transmembrane domain serine/threonine kinases, transcription factors, toll-like receptors and various other proteins (65). Although direct cause-function relationships between phosphorylation and regulation have not always been established, it is assumed that GRKs also have regulatory functions for these non-GPCR proteins. Second, GRKs can control signaling in a phosphorylation-independent manner *via* direct protein-protein interactions (50). GRK multifunctionality is probably best understood for GRK2, which can functionally interact with an extensive number of non-GPCR proteins due to its multidomain structure (16). These phosphorylation substrates and interactors of GRK2 also include proteins involved in cell adhesion and F-actin dynamics, which has mainly been discovered in studies with non-immune cells (66). Thus, we cannot rule out in our experiments with primary immune cells that the combined depletion of GRKs also interferes with non-GPCR effects or non-kinase activities of GRKs, which may also influence the migration properties of cells.

In fact, our experiments with DCs provide one such example where GRKs exert functions independent of ligand-activated GPCRs. While our initial *in vitro* and *in situ* analysis of *Grk2* ^−/−^/*Grk3* ^−/−^/*Grk6* ^−/−^ DCs revealed a CCR7-dependent migration deficit, more careful characterization of these cells uncovered an unexpected defect in DC development and maturation. We show that complete GRK deficiency impairs the homeostasis of classical DCs in spleen and lymph nodes, whereas a macrophage-like cell subset increases in numbers *in vivo*. This observation was also reflected in cultures of bone marrow precursors that develop to GM-DCs and GM-Macs in the presence of the growth and differentiation factor GM-CSF, which acts by activating the type I cytokine receptor CSF2R (30). Triple GRK knockout DCs shifted their *in vitro* development from GM-DCs toward GM-Macs, suggesting important roles of GRKs for DC specification. To our knowledge, there are no reports that the *in vitro* development of GM-DCs depends on GPCR function. Thus, it is very likely that the observed phenotype of triple knockout DCs relates to missing GRK functions other than phosphorylation of ligand-activated GPCRs. Perturbed balances of GM-CSF DC cultures and shifts toward macrophage development have previously been reported upon induced activation of the transcription factor NRF2 (67) or the depletion of the transcriptional zinc finger repressor GFI (68), the transcription factor NR4A3 (69), or several other regulators (70). A release of the DC restriction by the guanine nucleotide exchange factors DEF6 and SWAP70 may also underlie our observed phenotype (71). Thus, GRK deficiency may interfere with any of these of components, related transcriptional programs and signaling pathways, and the functional contribution of GRKs to these processes could involve kinase-dependent or –independent mechanisms. To address these questions, the profiling of the complete phospho-proteome in primary immune cells would be desirable. However, one needs to consider the technical challenges of such approaches and the limitations of their biological interpretation regarding direct and indirect effects of GRK-mediated phosphorylation events.

In summary, our study shows how the combined depletion of GRKs in immune cells provides a plethora of phenotypes, many of which are specific for an immune cell subset or GPCR type. Moreover, we demonstrate that combinatorial interference with GRK function alters immune cell processes, which go beyond GPCR desensitization. Together, our study highlights the need for studying GRK functions in primary immune cells to address their specific roles in each leukocyte subset.

## Data availability statement

The datasets presented in this study can be found in online repositories. The names of the repository/repositories and accession number(s) can be found below: https://www.ncbi.nlm.nih.gov/geo/, GSE212892.

## Ethics statement

The animal study was reviewed and approved by Regierungspräsidium Freiburg, Germany. Written informed consent was obtained from the owners for the participation of their animals in this study.

## Author contributions

KG and TL conceived and designed this project. KG performed the experiments and analyzed the results. TL supervised the project and acquired funding for the project. TT provided mice for the study. KG and TL wrote the manuscript. All authors read and approved the manuscript.

## Funding

This project was funded by an ERC-Starting Grant (715890) to TL and the Max Planck Society.

## Acknowledgments

We thank K. Ganter and F. Hormann for assistance with experiments and K. Kienle for initial technical help. We also thank the facilities for laboratory animals, imaging, flow cytometry, deep sequencing and bioinformatics at the Max Planck Institute of Immunobiology and Epigenetics for their support. Schematic illustrations were created with BioRender.com.

## Conflict of interest

The authors declare that the research was conducted in the absence of any commercial or financial relationships that could be construed as a potential conflict of interest.

## Publisher’s note

All claims expressed in this article are solely those of the authors and do not necessarily represent those of their affiliated organizations, or those of the publisher, the editors and the reviewers. Any product that may be evaluated in this article, or claim that may be made by its manufacturer, is not guaranteed or endorsed by the publisher.

## References

[1] Lämmermann T Kastenmüller W. Concepts of GPCR-controlled navigation in the immune system. Immunol Rev (2019) 289(1):205–31. doi: 10.1111/imr.12752 PMC6487968 30977203

[2] Steury MD McCabe LR Parameswaran N. G Protein-coupled receptor kinases in the inflammatory response and signaling. Adv Immunol (2017) 136:227–77. doi: 10.1016/bs.ai.2017.05.003 PMC5730335 28950947

[3] Freedman NJ Lefkowitz RJ. Desensitization of G protein-coupled receptors. Recent Prog Horm Res (1996) 51:319–51. discussion 52-3. 8701085

[4] Laganà M Schlecht-Louf G Bachelerie F. The G protein-coupled receptor kinases (GRKs) in chemokine receptor-mediated immune cell migration: From molecular cues to physiopathology. Cells (2021) 10(1):75. doi: 10.3390/cells10010075 33466410 PMC7824814

[5] Heng TSP Painter MW Elpek K Lukacs-Kornek V Mauermann N Turley SJ. The immunological genome project: networks of gene expression in immune cells. Nat Immunol (2008) 9(10):1091–4. doi: 10.1038/ni1008-1091 18800157

[6] Arnon TI Xu Y Lo C Pham T An J Coughlin S. GRK2-dependent S1PR1 desensitization is required for lymphocytes to overcome their attraction to blood. Science. (2011) 333(6051):1898–903. doi: 10.1126/science.1208248 PMC3267326 21960637

[7] Kienle K Glaser KM Eickhoff S Mihlan M Knöpper K Reátegui E. Neutrophils self-limit swarming to contain bacterial growth *in vivo* . Science (2021) 372(6548):eabe7729. doi: 10.1126/science.abe7729 34140358 PMC8926156

[8] Tarrant TK Billard MJ Timoshchenko RG McGinnis MW Serafin DS Foreman O. G Protein-coupled receptor kinase-3-deficient mice exhibit WHIM syndrome features and attenuated inflammatory responses. J Leukocyte Biol (2013) 94(6):1243–51. doi: 10.1189/jlb.0213097 10.1189/jlb.0213097 PMC3828605 23935208

[9] Petrie Aronin CE Zhao YM Yoon JS Morgan NY Prüstel T Germain RN. Migrating myeloid cells sense temporal dynamics of chemoattractant concentrations. Immunity (2017) 47(5):862–74.e3. doi: 10.1016/j.immuni.2017.10.020 29166587 PMC5726790

[10] Kavelaars A Vroon A Raatgever RP Fong AM Premont RT Patel DD. Increased acute inflammation, leukotriene B4-induced chemotaxis, and signaling in mice deficient for G protein-coupled receptor kinase 6. J Immunol (2003) 171(11):6128–34. doi: 10.4049/jimmunol.171.11.6128 14634128

[11] Raghuwanshi SK Su Y Singh V Haynes K Richmond A Richardson RM. The chemokine receptors CXCR1 and CXCR2 couple to distinct G protein-coupled receptor kinases to mediate and regulate leukocyte functions. J Immunol (2012) 189(6):2824–32. doi: 10.4049/jimmunol.1201114 PMC3436986 22869904

[12] Vroon A Heijnen CJ Raatgever R Touw IP Ploemacher RE Premont RT. GRK6 deficiency is associated with enhanced CXCR4-mediated neutrophil chemotaxis *in vitro* and impaired responsiveness to G-CSF *in vivo* . J Leukocyte Biol (2004) 75(4):698–704. doi: 10.1189/jlb.0703320 14704365

[13] Hwang IY Park C Harrison K Kehrl JH. Biased S1PR1 signaling in b cells subverts responses to homeostatic chemokines, severely disorganizing lymphoid organ architecture. J Immunol (2019) 203(9):2401–14. doi: 10.4049/jimmunol.1900678 PMC6814389 31548329

[14] Fong AM Premont RT Richardson RM Yu YR Lefkowitz RJ Patel DD. Defective lymphocyte chemotaxis in beta-arrestin2- and GRK6-deficient mice. Proc Natl Acad Sci USA (2002) 99(11):7478–83. doi: 10.1073/pnas.112198299 PMC124256 12032308

[15] Schwarz J Bierbaum V Vaahtomeri K Hauschild R Brown M de Vries I. Dendritic cells interpret haptotactic chemokine gradients in a manner governed by signal-to-Noise ratio and dependent on GRK6. Curr Biol (2017) 27(9):1314–25. doi: 10.1016/j.cub.2017.04.004 28457871

[16] Penela P Ribas C Sánchez-Madrid F Mayor F Jr. G Protein-coupled receptor kinase 2 (GRK2) as a multifunctional signaling hub. Cell Mol Life Sci (2019) 76(22):4423–46. doi: 10.1007/s00018-019-03274-3 PMC6841920 31432234

[17] Martini JS Raake P Vinge LE DeGeorge BR Jr. Chuprun JK Harris DM. Uncovering G protein-coupled receptor kinase-5 as a histone deacetylase kinase in the nucleus of cardiomyocytes. Proc Natl Acad Sci U S A. (2008) 105(34):12457–62. doi: 10.1073/pnas.0803153105 PMC2527933 18711143

[18] Zidar DA Violin JD Whalen EJ Lefkowitz RJ. Selective engagement of G protein coupled receptor kinases (GRKs) encodes distinct functions of biased ligands. Proc Natl Acad Sci USA. (2009) 106(24):9649–54. doi: 10.1073/pnas.0904361106 PMC2689814 19497875

[19] Nakai A Fujimoto J Miyata H Stumm R Narazaki M Schulz S. The COMMD3/8 complex determines GRK6 specificity for chemoattractant receptors. J Exp Med (2019) 216(7):1630–47. doi: 10.1084/jem.20181494 PMC6605747 31088898

[20] Matkovich SJ Diwan A Klanke JL Hammer DJ Marreez Y Odley AM. Cardiac-specific ablation of G-protein receptor kinase 2 redefines its roles in heart development and beta-adrenergic signaling. Circ Res (2006) 99(9):996–1003. doi: 10.1161/01.RES.0000247932.71270.2c 17008600

[21] Peppel K Boekhoff I McDonald P Breer H Caron MG Lefkowitz RJ. G Protein-coupled receptor kinase 3 (GRK3) gene disruption leads to loss of odorant receptor desensitization. J Biol Chem (1997) 272(41):25425–8. doi: 10.1074/jbc.272.41.25425 9325250

[22] Gainetdinov RR Bohn LM Walker JK Laporte SA Macrae AD Caron MG. Muscarinic supersensitivity and impaired receptor desensitization in G protein-coupled receptor kinase 5-deficient mice. Neuron (1999) 24(4):1029–36. doi: 10.1016/S0896-6273(00)81048-X 10624964

[23] Gainetdinov RR Bohn LM Sotnikova TD Cyr M Laakso A Macrae AD. Dopaminergic supersensitivity in G protein-coupled receptor kinase 6-deficient mice. Neuron (2003) 38(2):291–303. doi: 10.1016/S0896-6273(03)00192-2 12718862

[24] Passegué E Wagner EF Weissman IL. JunB deficiency leads to a myeloproliferative disorder arising from hematopoietic stem cells. Cell (2004) 119(3):431–43. doi: 10.1016/j.cell.2004.10.010 15507213

[25] de Boer J Williams A Skavdis G Harker N Coles M Tolaini M. Transgenic mice with hematopoietic and lymphoid specific expression of cre. Eur J Immunol (2003) 33(2):314–25. doi: 10.1002/immu.200310005 12548562

[26] Caton ML Smith-Raska MR Reizis B. Notch-RBP-J signaling controls the homeostasis of CD8- dendritic cells in the spleen. J Exp Med (2007) 204(7):1653–64. doi: 10.1084/jem.20062648 PMC2118632 17591855

[27] Rickert RC Roes J Rajewsky K. B lymphocyte-specific, cre-mediated mutagenesis in mice. Nucleic Acids Res (1997) 25(6):1317–8. doi: 10.1093/nar/25.6.1317 PMC146582 9092650

[28] Eberl G Littman DR. Thymic origin of intestinal alphabeta T cells revealed by fate mapping of RORgammat+ cells. Science (2004) 305(5681):248–51. doi: 10.1126/science.1096472 15247480

[29] Heit B Kubes P. Measuring chemotaxis and chemokinesis: the under-agarose cell migration assay. Sci STKE (2003) 2003(170):Pl5. doi: 10.1126/stke.2003.170.pl5 12591998

[30] Helft J Böttcher J Chakravarty P Zelenay S Huotari J Schraml BU. GM-CSF mouse bone marrow cultures comprise a heterogeneous population of CD11c(+)MHCII(+) macrophages and dendritic cells. Immunity (2015) 42(6):1197–211. doi: 10.1016/j.immuni.2015.05.018 26084029

[31] Liu Z Gu Y Chakarov S Bleriot C Kwok I Chen X. Fate mapping *via* Ms4a3-expression history traces monocyte-derived cells. Cell (2019) 178(6):1509–25.e19. doi: 10.1016/j.cell.2019.08.009 31491389

[32] Bhardwaj V Heyne S Sikora K Rabbani L Rauer M Kilpert F. snakePipes: facilitating flexible, scalable and integrative epigenomic analysis. Bioinformatics (2019) 35(22):4757–9. doi: 10.1093/bioinformatics/btz436 PMC6853707 31134269

[33] Dobin A Davis CA Schlesinger F Drenkow J Zaleski C Jha S. STAR: ultrafast universal RNA-seq aligner. Bioinformatics. (2013) 29(1):15–21. doi: 10.1093/bioinformatics/bts635 23104886 PMC3530905

[34] Liao Y Smyth GK Shi W. featureCounts: an efficient general purpose program for assigning sequence reads to genomic features. Bioinformatics (2014) 30(7):923–30. doi: 10.1093/bioinformatics/btt656 24227677

[35] Ramírez F Ryan DP Grüning B Bhardwaj V Kilpert F Richter AS. deepTools2: a next generation web server for deep-sequencing data analysis. Nucleic Acids Res (2016) 44(W1):W160–5. doi: 10.1093/nar/gkw257 PMC4987876 27079975

[36] Love MI Huber W Anders S. Moderated estimation of fold change and dispersion for RNA-seq data with DESeq2. Genome Biol (2014) 15(12):550. doi: 10.1186/s13059-014-0550-8 25516281 PMC4302049

[37] Mihlan M Glaser KM Epple MW Lämmermann T. Neutrophils: Amoeboid migration and swarming dynamics in tissues. Front Cell Dev Biol (2022) 10:871789. doi: 10.3389/fcell.2022.871789 35478973 PMC9038224

[38] Foxman EF Campbell JJ Butcher EC. Multistep navigation and the combinatorial control of leukocyte chemotaxis. J Cell Biol (1997) 139(5):1349–60. doi: 10.1083/jcb.139.5.1349 PMC2140208 9382879

[39] Masopust D Schenkel JM. The integration of T cell migration, differentiation and function. Nat Rev Immunol (2013) 13(5):309–20. doi: 10.1038/nri3442 23598650

[40] Lu E Cyster JG. G-Protein coupled receptors and ligands that organize humoral immune responses. Immunol Rev (2019) 289(1):158–72. doi: 10.1111/imr.12743 PMC6464390 30977196

[41] Cabeza-Cabrerizo M Cardoso A Minutti CM MPd C. Sousa CRe. dendritic cells revisited. Annu Rev Immunol (2021) 39(1):131–66. doi: 10.1146/annurev-immunol-061020-053707 33481643

[42] Miller JC Brown BD Shay T Gautier EL Jojic V Cohain A. Deciphering the transcriptional network of the dendritic cell lineage. Nat Immunol (2012) 13(9):888–99. doi: 10.1038/ni.2370 PMC3985403 22797772

[43] Gautier EL Shay T Miller J Greter M Jakubzick C Ivanov S. Gene-expression profiles and transcriptional regulatory pathways that underlie the identity and diversity of mouse tissue macrophages. Nat Immunol (2012) 13(11):1118–28. doi: 10.1038/ni.2419 PMC3558276 23023392

[44] Calabro S Liu D Gallman A Nascimento MS Yu Z Zhang TT. Differential intrasplenic migration of dendritic cell subsets tailors adaptive immunity. Cell Rep (2016) 16(9):2472–85. doi: 10.1016/j.celrep.2016.07.076 PMC6323650 27545885

[45] Chistiakov DA Killingsworth MC Myasoedova VA Orekhov AN Bobryshev YV. CD68/macrosialin: not just a histochemical marker. Lab Invest. (2017) 97(1):4–13. doi: 10.1038/labinvest.2016.116 27869795

[46] Arveseth CD Happ JT Hedeen DS Zhu JF Capener JL Klatt Shaw D. Smoothened transduces hedgehog signals *via* activity-dependent sequestration of PKA catalytic subunits. PloS Biol (2021) 19(4):e3001191. doi: 10.1371/journal.pbio.3001191 33886552 PMC8096101

[47] Drube J Haider RS Matthees ESF Reichel M Zeiner J Fritzwanker S. GPCR kinase knockout cells reveal the impact of individual GRKs on arrestin binding and GPCR regulation. Nat Commun (2022) 13(1):540. doi: 10.1038/s41467-022-28152-8 35087057 PMC8795447

[48] Pandey S Kumari P Baidya M Kise R Cao Y Dwivedi-Agnihotri H. Intrinsic bias at non-canonical, β-arrestin-coupled seven transmembrane receptors. Mol Cell (2021) 81(22):4605–21.e11. doi: 10.1016/j.molcel.2021.09.007 34582793 PMC7612807

[49] Franco A Zhang L Matkovich SJ Kovacs A Dorn GW 2nd. G-Protein receptor kinases 2, 5 and 6 redundantly modulate smoothened-GATA transcriptional crosstalk in fetal mouse hearts. J Mol Cell Cardiol (2018) 121:60–8. doi: 10.1016/j.yjmcc.2018.06.009 PMC6178805 29969579

[50] Gurevich VV Gurevich EV. GPCR signaling regulation: The role of GRKs and arrestins. Front Pharmacol (2019) 10:125. doi: 10.3389/fphar.2019.00125 30837883 PMC6389790

[51] Prihandoko R Bradley SJ Tobin AB Butcher AJ. Determination of GPCR phosphorylation status: Establishing a phosphorylation barcode. Curr Protoc Pharmacol (2015) 69:2.13.1-2.13.26. doi: 10.1002/0471141755.ph0213s69 26344213

[52] Doll C Konietzko J Pöll F Koch T Höllt V Schulz S. Agonist-selective patterns of µ-opioid receptor phosphorylation revealed by phosphosite-specific antibodies. Br J Pharmacol (2011) 164(2):298–307. doi: 10.1111/j.1476-5381.2011.01382.x 21449911 PMC3174411

[53] Tobin AB Butcher AJ Kong KC. Location, location, location.site-specific GPCR phosphorylation offers a mechanism for cell-type-specific signalling. Trends Pharmacol Sci (2008) 29(8):413–20. doi: 10.1016/j.tips.2008.05.006 PMC2880250 18606460

[54] Busillo JM Armando S Sengupta R Meucci O Bouvier M Benovic JL. Site-specific phosphorylation of CXCR4 is dynamically regulated by multiple kinases and results in differential modulation of CXCR4 signaling. J Biol Chem (2010) 285(10):7805–17. doi: 10.1074/jbc.M109.091173 PMC2844224 20048153

[55] Mueller W Schütz D Nagel F Schulz S Stumm R. Hierarchical organization of multi-site phosphorylation at the CXCR4 c terminus. PloS One (2013) 8(5):e64975. doi: 10.1371/journal.pone.0064975 23734232 PMC3666969

[56] Kohout TA Nicholas SL Perry SJ Reinhart G Junger S Struthers RS. Differential desensitization, receptor phosphorylation, beta-arrestin recruitment, and ERK1/2 activation by the two endogenous ligands for the CC chemokine receptor 7. J Biol Chem (2004) 279(22):23214–22. doi: 10.1074/jbc.M402125200 15054093

[57] O'Sullivan C Dev KK. The structure and function of the S1P1 receptor. Trends Pharmacol Sci (2013) 34(7):401–12. doi: 10.1016/j.tips.2013.05.002 23763867

[58] Otero C Eisele PS Schaeuble K Groettrup M Legler DF. Distinct motifs in the chemokine receptor CCR7 regulate signal transduction, receptor trafficking and chemotaxis. J Cell Sci (2008) 121(Pt 16):2759–67. doi: 10.1242/jcs.029074 18664492

[59] Watterson KR Johnston E Chalmers C Pronin A Cook SJ Benovic JL. Dual regulation of EDG1/S1P(1) receptor phosphorylation and internalization by protein kinase c and G-protein-coupled receptor kinase 2. J Biol Chem (2002) 277(8):5767–77. doi: 10.1074/jbc.M110647200 11741892

[60] Butcher AJ Prihandoko R Kong KC McWilliams P Edwards JM Bottrill A. Differential G-protein-coupled receptor phosphorylation provides evidence for a signaling bar code. J Biol Chem (2011) 286(13):11506–18. doi: 10.1074/jbc.M110.154526 PMC3064205 21177246

[61] Matthees ESF Haider RS Hoffmann C Drube J. Differential regulation of GPCRs-are GRK expression levels the key? Front Cell Dev Biol (2021) 9:687489. doi: 10.3389/fcell.2021.687489 34109182 PMC8182058

[62] Nobles KN Xiao K Ahn S Shukla AK Lam CM Rajagopal S. Distinct phosphorylation sites on the β(2)-adrenergic receptor establish a barcode that encodes differential functions of β-arrestin. Sci Signal (2011) 4(185):ra51. doi: 10.1126/scisignal.2001707 21868357 PMC3415961

[63] Torrecilla I Spragg EJ Poulin B McWilliams PJ Mistry SC Blaukat A. Phosphorylation and regulation of a G protein-coupled receptor by protein kinase CK2. J Cell Biol (2007) 177(1):127–37. doi: 10.1083/jcb.200610018 PMC2064117 17403928

[64] Violin JD Ren XR Lefkowitz RJ. G-Protein-coupled receptor kinase specificity for beta-arrestin recruitment to the beta2-adrenergic receptor revealed by fluorescence resonance energy transfer. J Biol Chem (2006) 281(29):20577–88. doi: 10.1074/jbc.M513605200 16687412

[65] Gurevich EV Tesmer JJ Mushegian A Gurevich VV. G Protein-coupled receptor kinases: more than just kinases and not only for GPCRs. Pharmacol Ther (2012) 133(1):40–69. doi: 10.1016/j.pharmthera.2011.08.001 21903131 PMC3241883

[66] Penela P Nogués L Mayor F Jr. Role of G protein-coupled receptor kinases in cell migration. Curr Opin Cell Biol (2014) 27:10–7. doi: 10.1016/j.ceb.2013.10.005 24680425

[67] Vallelian F Buzzi RM Pfefferlé M Yalamanoglu A Dubach IL Wassmer A. Heme-stress activated NRF2 skews fate trajectories of bone marrow cells from dendritic cells towards red pulp-like macrophages in hemolytic anemia. Cell Death Differ (2022) 29(8):1450–65. doi: 10.1038/s41418-022-00932-1 PMC9345992 35031770

[68] Rathinam C Geffers R Yücel R Buer J Welte K Möröy T. The transcriptional repressor Gfi1 controls STAT3-dependent dendritic cell development and function. Immunity (2005) 22(6):717–28. doi: 10.1016/j.immuni.2005.04.007 15963786

[69] Boulet S Daudelin JF Odagiu L Pelletier AN Yun TJ Lesage S. The orphan nuclear receptor NR4A3 controls the differentiation of monocyte-derived dendritic cells following microbial stimulation. Proc Natl Acad Sci USA (2019) 116(30):15150–9. doi: 10.1073/pnas.1821296116 PMC6660778 31285338

[70] Anderson DA 3rd Dutertre CA Ginhoux F Murphy KM. Genetic models of human and mouse dendritic cell development and function. Nat Rev Immunol (2021) 21(2):101–15. doi: 10.1038/s41577-020-00413-x PMC10955724 32908299

[71] Popović J Wellstein I Pernis A Jessberger R Ocaña-Morgner C. Control of GM-CSF-Dependent dendritic cell differentiation and maturation by DEF6 and SWAP-70. J Immunol (2020) 205(5):1306–17. doi: 10.4049/jimmunol.2000020 32709659

