# Supplementary material for: Combinatorial depletions of G-protein coupled receptor kinases in immune cells identify pleiotropic and cell type-specific functions
Source: Front Immunol. 2022 Nov 14;13:1039803. doi: 10.3389/fimmu.2022.1039803 (PMC9703078; doi:10.3389/fimmu.2022.1039803)
Supplement: Supplementary file 1 [file DataSheet_1.pdf]

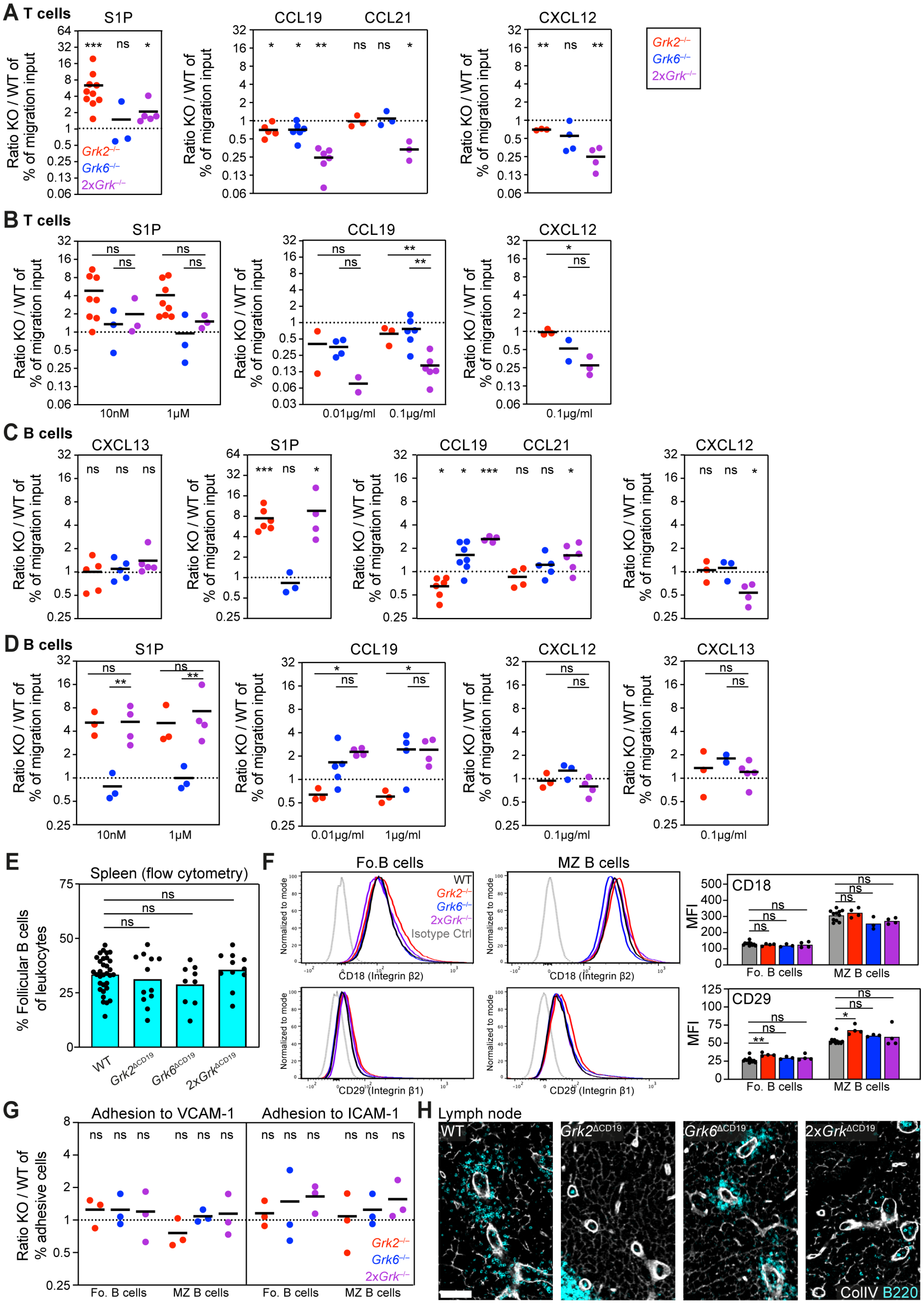

Supplementary Figure 1: Roles of GRKs for T cell chemotaxis, and B cell chemotaxis, numbers and positioning. (A) Transwell migration of splenic T cells, data taken from Fig.2 D-F. *n* = 3-8 biological replicates performed as independent experiments with *N* = 2 technical replicates each. Bars display the

mean. ****P*<0.001, ***P*<0.01, **P*<0.05, ns nonsignificant, one sample *t* test. **(B)** Transwell migration of splenic T cells toward 10 nM and 1 µM S1P, 0.01 µg/ml and 0.1 µg/ml CCL19 and 0.1 µg/ml CXCL12. Displayed are ratios of KO versus WT. *n* = 2-8 biological replicates performed as independent experiments with *N* = 2 technical replicates each. Bars display the mean. ***P*<0.01, **P*<0.05, ns nonsignificant, post hoc after ANOVA. **(C)** Transwell migration of splenic B cells, data taken from Fig.3 C-F. *n* = 3-7 biological replicates performed as independent experiments with *N* = 2 technical replicates each. Bars display the mean. ****P*<0.001, ***P*<0.01, **P*<0.05, ns nonsignificant, one sample *t* test. **(D)** Transwell migration of splenic B cells toward 10 nM and 1 µM S1P, 0.01 µg/ml and 1 µg/ml CCL19 and 0.1 µg/ml CXCL12. Displayed are ratios of KO versus WT. *n* = 2-5 biological replicates performed as independent experiments with *N* = 2 technical replicates each. Bars display the mean. ***P*<0.01, **P*<0.05, ns nonsignificant, post hoc after ANOVA. **(E)** Frequency of follicular B cells compared to all leukocytes, measured by flow cytometry; n = 9-12 mice per gene knockout. Bars display the mean. ns nonsignificant, post hoc after ANOVA. **(F)** Expression of CD18 (Integrin ß1, upper row) and CD29 (Integrin ß1, lower row) on follicular (Fo.) and marginal zone (MZ) B cells as measured by flow cytometry. Histograms of representative experiments (left and middle), and quantification (right). *n* = 3-4 mice per genotype. Bars display the mean. ***P*<0.01, **P*<0.05, ns nonsignificant, post hoc after ANOVA (CD18) or Kruskal-Wallis (CD29). **(G)** Adhesion of follicular (Fo.) and marginal zone (MZ) B cells to VCAM-1 and ICAM-1 upon stimulation with 1 µg/ml CXCL12 and 1 µg/ml CXCL13. Displayed are ratios of KO versus WT of % adhering cells of input. *n* = 3 mice per genotype with *N* = 2 technical replicates each. Bars display the mean. ns nonsignificant, one sample *t* test. **(H)** Positioning of WT and *Grk*-deficient B cells in proximity to high endothelial venules in homeostatic lymph nodes. B cells were detected with immunostaining against B220 (cyan), HEVs with Collagen IV (white). Scale bar: 70 µm.

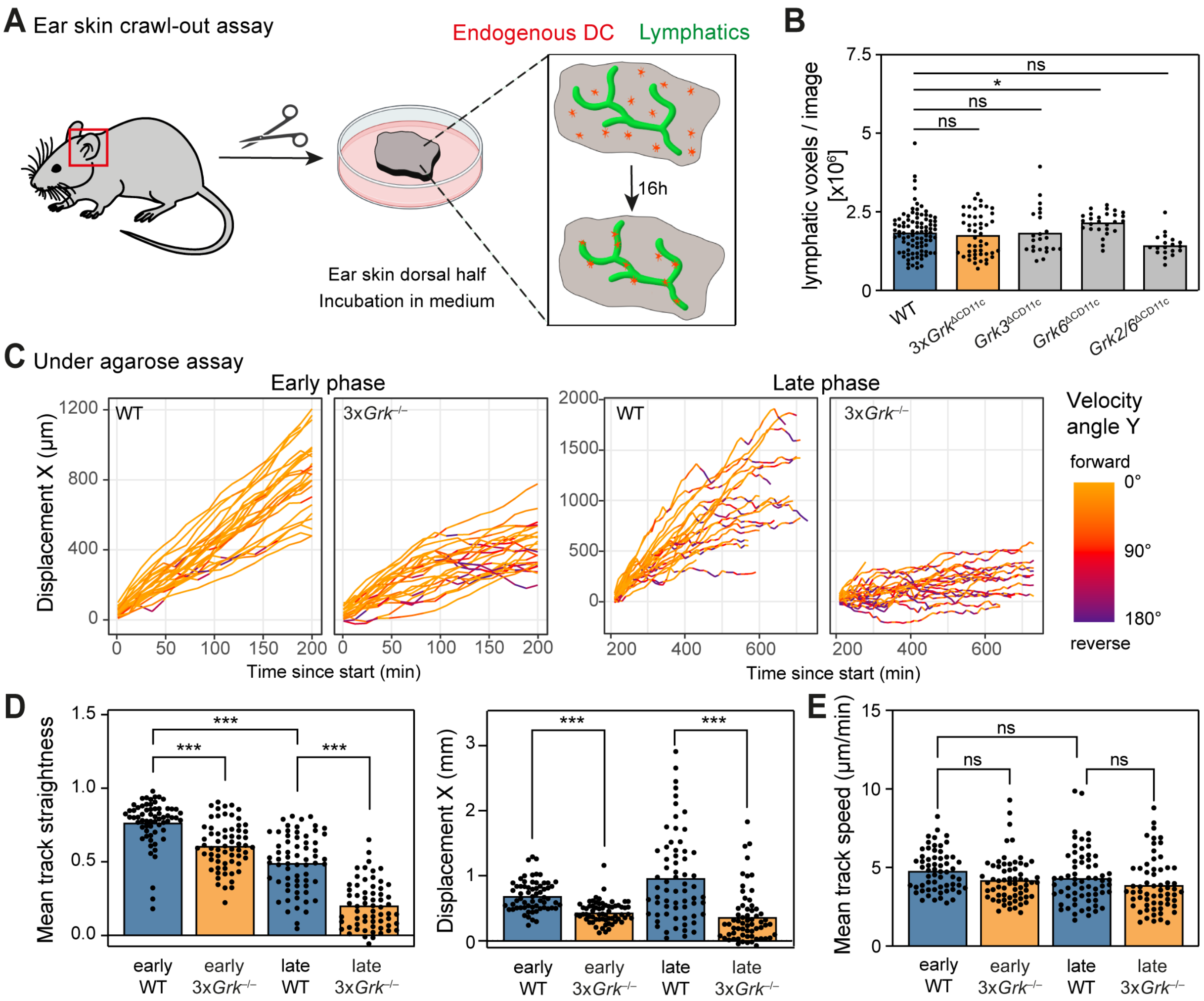

Supplementary Figure 2: Analysis of DC migration *in situ* and *in vitro*. (A) Scheme of the ear skin crawl-out assay. Ears are split into two halves, the dorsal part is incubated floating on medium. Within 16 h skin DCs are activated and triggered to migrate into the lymphatic vessels. **(B)** Quality control of analyzed images for Fig. 4D, quantification of lymphatic vessels. Bars display the mean. ns nonsignificant, post hoc after Kruskal-Wallis. **(C)** Analysis of DC chemotaxis in the under-agarose assay, categorized into early (0-3 h after start of experiment) and late (3-12 h) phases. Plotted is the displacement toward the attractor well normalized to the start point of the track (early phase) or the position after 3 h (late phase). The velocity angle Y is color-coded and indicates forward (0°-90°, orange) and reverse (90°-180°, purple) migration. Representative experiment is also shown in Fig. 4I, K. **(D, E)** Quantification of DC mean track straightness and displacement towards attractor hole (along x axis) (D) and mean track speed (E), analyzed for early and late phases. Bars display the mean. ***$P<0.001$, ns nonsignificant, post hoc after Kruskal-Wallis test (D) or ANOVA (E).

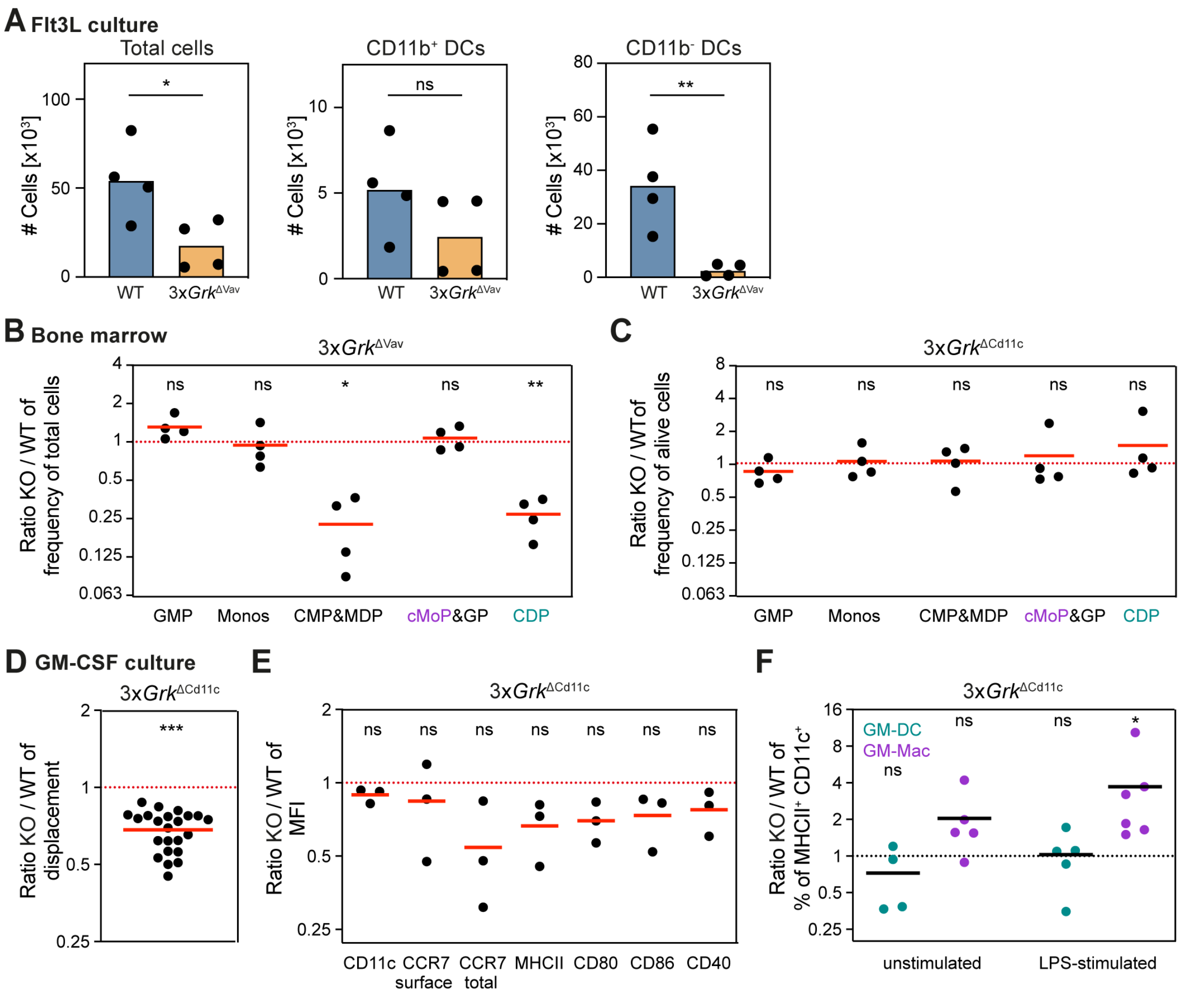

Supplementary Figure 3: Analysis of DC development *in vitro*. **(A)** Quantification of DC populations in Flt3L bone marrow cultures of WT or 3x$Grk^{\Delta Vav}$ mice. Total cell number per culture dish, CD11c$^+$ CD11b$^-$ cDCs and CD11c$^+$ CD11b$^+$ cDCs. n = 2 biological replicates with N = 2 technical replicates. Bars display the mean. **P<0.01, *P<0.05, ns nonsignificant, one sample t test (total cells, CD11b$^-$ DCs) or Mann-Whitney test (CD11b$^+$ DCs). **(B,C)** Quantification of leukocyte progenitors in the bone marrow of the 3x$Grk^{\Delta Vav}$ (B) or 3x$Grk^{\Delta CD11c}$ (C) mouse model. Displayed are ratios 3x$Grk^{-/-}$ versus WT. n = 4 mice per genotype. Bars display the mean. **P<0.01, *P<0.05, ns nonsignificant, one sample t test. **(D)** Endpoint displacement of BMDCs generated from 3x$Grk^{\Delta CD11c}$ vs. BMDCs from WT mice in the under agarose assay (refers to Fig. 4L). n = 3 biological replicates performed as independent experiments with N = 7-8 technical repeats per experiment. Bar displays the mean. ***P<0.001, one sample t test. **(E)** Quantification of DC marker expression levels in CD11c$^+$ LPS-stimulated BMDCs (refers to Fig. 5C). Displayed are ratios of BMDCs generated from 3x$Grk^{\Delta CD11c}$ vs. BMDCs from WT mice. n = 3 biological replicates performed as independent experiments. Bars display the mean. ns nonsignificant, one sample t test. **(F)** Quantification of bona fide DCs (GM-DC) and macrophages (GM-Mac) developing in GM-CSF cultures (refers to Fig. 5E). Displayed are ratios BMDCs generated from 3x$Grk^{\Delta CD11c}$ vs. BMDCs from WT mice with or without LPS stimulation. n = 4-6 biological replicates performed as independent experiments. Bars display the mean. *P<0.05, ns nonsignificant, one sample t test.

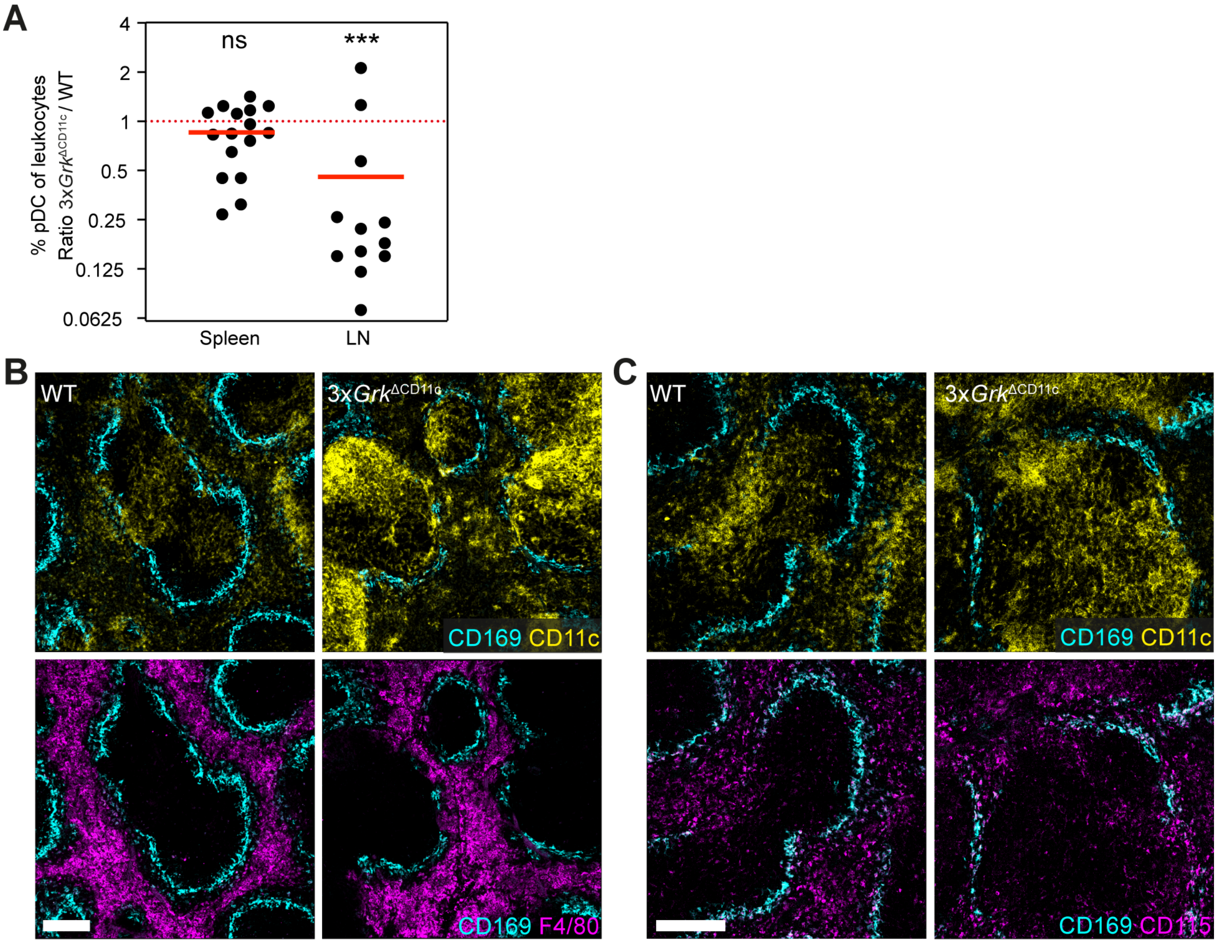

Supplementary Figure 4: Analysis of pDC development and macrophage-like cells in lymphoid organs. (A) Quantification of pDCs in spleens and lymph nodes compared to all leukocytes by flow cytometry. Displayed are ratios of 3x*Grk*$^{\Delta CD11c}$ versus WT. *n* = 12-16 mice per genotype. Bars display the mean. ***P<0.001, ns nonsignificant, one sample *t* test. **(B, C)** Representative immunofluorescence images comparing F4/80 (B) and CD115 (C) expression (magenta) in spleens of 3x*Grk*$^{\Delta CD11c}$ mice and littermate controls. Marginal zone macrophages (CD169, cyan) and CD11c (yellow) are displayed for orientation. Scale bars: 100 µm.
