# Supplementary material for: Combinatorial depletions of G-protein coupled receptor kinases in immune cells identify pleiotropic and cell type-specific functions
Source: Front Immunol. 2022 Nov 14;13:1039803. doi: 10.3389/fimmu.2022.1039803 (PMC9703078; doi:10.3389/fimmu.2022.1039803)
Supplement: Supplementary file 2 [file DataSheet_2.pdf]

Supplementary Tables

Supplementary Table 1. List of antibodies used for flow cytometry (FC), immunoblotting (WB) and immunofluorescence (IF).

Target	Clone	Labeling	Company	Catalog number	Assay
33D1	33D1	biotin	BioLegend	124903	IF
B220	RA3-6B2	APC-Cy7	BioLegend	103223	FC
B220	RA3-6B2	PE-Cy7	BioLegend	103221	FC
B220	RA3-6B2	A647	BioLegend	103226	FC, IF
B220	RA3-6B2	BV785	BioLegend	103245	FC
CCR7 (CD197)	4B12	PE	BioLegend	120106	FC
CD3	17A2	APC-Cy7	BioLegend	100221	FC
CD3	145-2C11	FITC	BioLegend	100306	FC
CD3	145-2C11	PE	invitrogen	12-0031-83	IF
CD11b	M1/70	A488	BioLegend	101217	FC
CD11b	M1/70	BUV385	BDBiosciences	563553	FC
CD11c	N418	APC-Cy7	BioLegend	117324	FC
CD11c	N418	A488	BioLegend	117311	FC
CD11c	N418	PE	BioLegend	117307	FC, IF
CD16/32	93	BV510	BioLegend	101333	FC
CD18	C71/16	PE	BD	553293	FC
CD19	6D5	APC-Cy7	BioLegend	115530	FC
CD19	eBio1D3	PE-Cy7	invitrogen	25-0193-81	FC
CD21	7E9	PE-Cy7	BioLegend	123420	FC
CD21	7E9	PE	BioLegend	123410	FC
CD23	B3B4	FITC	BioLegend	101606	FC
CD23	B3B4	BV421	BioLegend	101621	FC
CD29	HMb1-1	APC	BioLegend	102215	FC
CD34	SA376A4	BV421	BioLegend	152207	FC
CD40	3/23	APC-Fire/750	BioLegend	124631	FC
CD45.1	A20	BV510	BioLegend	110741	FC
CD45.2	104	BV711	BioLegend	109847	FC
CD64	X54-5/7.1	BV421	BioLegend	139309	FC
CD68	FA-11	eF660	eBioscience	50-0681-80	IF
CD80	16-10A1	BV421	BioLegend	104725	FC
CD86	GL-1	PE-Cy7	BioLegend	105013	FC
CD115	AFS98	BV421	BioLegend	135513	FC
CD115	AFS98	BV605	BioLegend	135517	FC
CD115	AFS98	PE	eBioscience	12-1152-81	FC, IF
CD117	2B8	BV421	BioLegend	105827	FC
CD117	2B8	PE	eBioscience	12-1171-83	FC
CD135 (Flt3)	A2F10	Biotin	BioLegend	135307	FC
CD135 (Flt3)	A2F10	PE	BioLegend	135305	FC
CD169	3D6.112	A488	BioLegend	142419	IF
CD172a (SIRP1a)	P84	APC	BioLegend	144013	FC
CD370 (DNGR-1)	10B4	BV510	BDBiosciences	744510	FC
Collagen IV	polyclonal	-	abcam	ab19808	IF

F4/80	BM8	BV421	BioLegend	123137	IF
GRK2	C-9	-	SCBT	Sc-13143	WB
GRK3	Polyclonal	-	SCBT	Sc-653	WB
GRK6	Polyclonal	-	SCBT	Sc-656	WB
Ly6C	HK1.4	A647	BioLegend	128010	FC
Ly6C	HK1.4	BV785	BioLegend	128041	FC
Ly6C	HK1.4	A488	BioLegend	128021	FC
Ly6C	HK1.4	APC-Cy7	BioLegend	128025	FC
Ly6G	1A8	APC-Cy7	BioLegend	127624	FC
Lyve-1	ALY7	A488	eBioscience	53-0443-80	IF
MCH-II	M5/114.15.2	APC-Cy7	BioLegend	107628	FC
MCH-II	M5/114.15.2	FITC	BioLegend	107605	FC
MCH-II	M5/114.15.2	BV510	BioLegend	107635	FC
MCH-II	M5/114.15.2	BV711	BioLegend	107643	FC
MHCII	M5/114.15.2	A594	BioLegend	107650	IF
MerTK	2B10C42	APC	BioLegend	151507	FC
MerTK	polyclonal	Biotin	R&D systems	BAF591	FC, IF
NK1.1	PK136	APC-Cy7	BioLegend	108723	FC
Sca-1	D7	APC-Cy7	BioLegend	108125	FC
Siglec-H	551	FITC	BioLegend	129603	FC
Ter119	TER-119	APC-Cy7	BioLegend	116223	FC
XCR1	ZET	PE	BioLegend	148204	FC
XCR1	ZET	A647	BioLegend	148213	IF
2nd a-rb	Polyclonal	A405	Invitrogen	A31556	IF
2nd a-rb	Polyclonal	PacO	Invitrogen	P31584	IF
2nd a-ms	Polyclonal	HRP	Dako	P0161	WB
2nd a-rb	Polyclonal	HRP	Dako	P0217	WB
Streptavidin	-	PE-Cy7	BioLegend	405206	FC
Streptavidin	-	BV421	BioLegend	405226	IF
Isotype control Hamster IgG	HTK888	A647	BioLegend	400924	FC
Isotype control Rat IgG2a κ	R35-95	PE	BD	553930	FC

Supplementary Table 2. List of primers used for RT-qPCR to determine the efficiencies of conditional knockouts in T- and B-cells.

**Gene**	**Primers (forward; reverse)**
Grk2	5'-GGCCTGCTCACATCCCTTTT-3'; 5'-CTTCTGGAACACGTCCCCTC-3'
Grk6	5'-GTAGCGAACACGGTGCTACT-3'; 5'-GTCACGCTCAAGGCTGAGT-3'
Actin	5'-GGCTGTATTCCCCTCCATCG-3'; 5'-CCAGTTGGTAACAATGCCATGT-3'
Gapdh	5'-AGGTCGGTGTGAACGGATTTG-3'; 5'-TGTAGACCATGTAGTTGAGGTCA-3'

Supplementary Table 3. Summary of statistical tests.

**Figure**	**Statist. test**		**Posthoc test**	
**1D**<br>CXCL2<br>LTB4	One sample *t*	 *t*=14.05, *df*=5, *P*<0.0001<br>*t*=8.569, *df*=5 *P*=0.0004		 ***<br>***
**1G left**<br>Wm<br>WM<br>C5a	One sample *t*	 *t*=8.084, *df*=4, *P*=0.0022<br>*t*=7.564, *df*=3 *P*=0.0072<br>*t*=9.090, *df*=5 *P*=0.0009		 **<br>**<br>***
**1G right**<br>Wm<br>WM<br>C5a	*t* test	 *t*=3.856, *df*=45, *P*=0.0004<br>*t*=4.626, *df*=45, *P*<0.0001<br>*t*=6.124, *df*=45, *P*<0.0001		 ***<br>***<br>***
**2D**	ANOVA	*F*=7.328, *DFn*=2, *DFd*=15, *P*=0.0060	2x$Grk^{-/-}$:$Grk2^{-/-}$ *P*=0.0376<br>2x$Grk^{-/-}$:$Grk6^{-/-}$ *P*=0.4282	*<br>ns
**2E CCL19**	ANOVA	*F*=14.96, *DFn*=2, *DFd*=14, *P*=0.0200	2x$Grk^{-/-}$:$Grk2^{-/-}$ *P*=0.0008<br>2x$Grk^{-/-}$:$Grk6^{-/-}$ *P*=0.0006	***<br>***
**2E CCL21**	ANOVA	*F*=15.54, *DFn*=2, *DFd*=6, *P*=0.0042	2x$Grk^{-/-}$:$Grk2^{-/-}$ *P*=0.0065<br>2x$Grk^{-/-}$:$Grk6^{-/-}$ *P*=0.0043	**<br>**
**2F**	ANOVA	*F*=6.255, *DFn*=2, *DFd*=8, *P*=0.0231	2x$Grk^{-/-}$:$Grk2^{-/-}$ *P*=0.0177<br>2x$Grk^{-/-}$:$Grk6^{-/-}$ *P*=0.0660	*<br>ns
**2G**	ANOVA	*F*=7.528, *DFn*=3, *DFd*=32, *P*=0.0006	WT:2x$Grk^{-/-}$ *P*=0.0003<br>WT:$Grk2^{-/-}$ *P*=0.0816<br>WT:$Grk6^{-/-}$ *P*>0.9999	***<br>ns<br>ns
**2I**<br>$Grk2^{-/-}$<br>$Grk6^{-/-}$<br>2x$Grk^{-/-}$	One sample *t* test	 *t*=6.773, *df*=5, *P*=0.0005<br>*t*=0.4215, *df*=2, *P*=0.7144<br>*t*=2.833, *df*=5, *P*=0.0366		 ***<br>ns<br>*
**3C**	ANOVA	*F*=1.179, *DFn*=2, *DFd*=12, *P*=0.3410	2x$Grk^{-/-}$:$Grk2^{-/-}$ *P*=0.2565<br>2x$Grk^{-/-}$:$Grk6^{-/-}$ *P*=0.5713	ns<br>ns
**3D**	ANOVA	*F*=21.01, *DFn*=2, *DFd*=10, *P*=0.0003	2x$Grk^{-/-}$:$Grk2^{-/-}$ *P*=0.9505<br>2x$Grk^{-/-}$:$Grk6^{-/-}$ *P*=0.0004	ns<br>***
**3E CCL19**	ANOVA	*F*=24.04, *DFn*=2, *DFd*=14, *P*<0.0001	2x$Grk^{-/-}$:$Grk2^{-/-}$ *P*<0.0001<br>2x$Grk^{-/-}$:$Grk6^{-/-}$ *P*=0.0356	***<br>*
**3E CCL21**	ANOVA	*F*=3.706, *DFn*=2, *DFd*=12, *P*=0.0558	2x$Grk^{-/-}$:$Grk2^{-/-}$ *P*=0.0344<br>2x$Grk^{-/-}$:$Grk6^{-/-}$ *P*=0.3849	*<br>ns
**3F**	ANOVA	*F*=6.051, *DFn*=2, *DFd*=7, *P*=0.0298	2x$Grk^{-/-}$:$Grk2^{-/-}$ *P*=0.0483<br>2x$Grk^{-/-}$:$Grk6^{-/-}$ *P*=0.0326	*<br>*
**3G**	ANOVA	*F*=0.7123, *DFn*=3, *DFd*=66, *P*=0.7123	WT:2x$Grk^{-/-}$ *P*=0.9327<br>WT:$Grk2^{-/-}$ *P*=0.8259<br>WT:$Grk6^{-/-}$ *P*=0.7298	ns<br>ns<br>ns
**3K**<br>2x$Grk^{-/-}$<br>$Grk2^{-/-}$<br>$Grk6^{-/-}$	One sample *t* test	 *t*=3.617, *df*=12, *P*=0.0035<br>*t*=4.371, *df*=8, *P*=0.0024<br>*t*=4.928, *df*=10, *P*=0.0006		 **<br>**<br>***
**3L**<br>$Grk2^{-/-}$<br>$Grk6^{-/-}$<br>2x$Grk^{-/-}$	One sample *t* test	 *t*=10.01 *df*=3, *P*=0.0021<br>*t*=0.2578, *df*=4, *P*=0.8093<br>*t*=4.521, *df*=8, *P*=0.0019		 **<br>ns<br>**
**4D**	Kruskal–Wallis	*H*(5)=92.12, *P*<0.0001	WT:3x$Grk^{-/-}$ *P*<0.0001<br>WT:2x$Grk^{-/-}$ *P*>0.9999<br>WT:$Grk3^{-/-}$ *P*>0.9999<br>WT:$Grk6^{-/-}$ *P*>0.9999	***<br>ns<br>ns<br>ns
**4E**	One sample *t* test	*t*=0.3929, *df*=6, *P*=0.7080		ns

**4F**<br>3x*Grk*$^{-/-}$<br>2x*Grk*$^{-/-}$<br>*Grk3*$^{-/-}$<br>*Grk6*$^{-/-}$	One sample *t* test	 t=7.988, df=16, P<0.0001<br>t=0.1047, df=3, P=0.9233<br>t=0.1612, df=4, P=0.8797<br>t=0.0121, df=3, P=0.9911		 ***<br>ns<br>ns<br>ns
**4K**	*t* test	t=0.5172, df=127,<br>P=0.6059		ns
**4L**	ANOVA	F=28.55, DFn=6,<br>DFd=124, P<0.0001	3x*Grk*$^{-/-}$:2x*Grk*$^{-/-}$ P=0.3091<br>3x*Grk*$^{-/-}$:*Grk2*$^{-/-}$ P=0.0029	ns<br>**
**4M**	One sample *t* test	t=9.845, df=19, P<0.0001		***
**5B**<br>Surface<br>Total	One sample *t* test	 t=96.265, df=7, P=0.0004<br>t=6.572, df=2, P=0.0224		 ***<br>*
**5C**<br>CD11c<br>MHCII<br>CD80<br>CD86<br>CD40	One sample *t* test	 t=0.0904, df=9, P=0.9299<br>t=6.064, df=5, P=0.0018<br>t=3.309, df=9, P=0.0091<br>t=4.329, df=9, P=0.0019<br>t=3.072, df=4, P=0.0372		 ns<br>**<br>**<br>**<br>*
**5E**<br>DC unstim<br>Mac unstim<br>DC stim<br>Mac stim	One sample *t* test	 t=5.898, df=5, P=0.0020<br>t=4.607, df=5, P=0.0058<br>t=2.339, df=6, P=0.0580<br>t=4.607, df=6, P=0.0058		 **<br>**<br>ns<br>**
**5F**<br>CCR7<br>MHCII<br>CD40	One sample *t* test	 t=5.539, df=5, P=0.0026<br>t=2.889, df=5, P=0.0342<br>t=2.421, df=5, P=0.0601		 **<br>*<br>ns
**5H left**<br>CDP<br>cMoP<br>MDP	 *t* test<br>Mann-Whitney<br>*t* test	 t=1.017, df=6, P=0.3484<br>P=0.0286<br>t=3.389, df=4, P=0.0276		 ns<br>*<br>*
**5H right**<br>CDP<br>cMoP<br>MDP	One sample *t* test	 t=1.672, df=3, P=0.1932<br>t=4.487, df=3, P=0.0207<br>t=4.985, df=2, P=0.0380		 ns<br>*<br>*
**5I**<br>CDP-DC<br>cMoP-Mac<br>MDP-DC<br>MDP-Mac	One sample *t* test	 t=1.663, df=3, P=0.1948<br>t=29.98, df=3, P=0.0001<br>t=2.983, df=2, P=0.0964<br>t=14.65, df=2, P=0.0046		 ns<br>***<br>ns<br>**
**6C**<br>cDC1<br>cDC2	One sample *t* test	 t=3.765, df=7, P=0.0070<br>t=3.164, df=7, P=0.0158		 **<br>*
**7B**<br>cDC1<br>cDC2	One sample *t* test	 t=0.7704, df=6, P=0.4703<br>t=2.123, df=6, P=0.0780		 ns<br>ns
**S1A S1P**<br>*Grk2*$^{-/-}$<br>*Grk6*$^{-/-}$<br>2x*Grk*$^{-/-}$	One sample *t* test	 t=7.166, df=9 P<0.0001<br>t=0.1035, df=2 P=0.9270<br>t=3.261, df=4 P=0.0311		 ***<br>ns<br>*
**S1A CCL19**<br>*Grk2*$^{-/-}$<br>*Grk6*$^{-/-}$<br>2x*Grk*$^{-/-}$	One sample *t* test	 t=3.161, df=4, P=0.0341<br>t=2.842, df=5 P=0.0362<br>t=6.658, df=5 P=0.0012		 *<br>*<br>**

**S1A CCL21**<br>*Grk2*$^{-/-}$<br>*Grk6*$^{-/-}$<br>2x*Grk*$^{-/-}$	One sample *t* test	 *t*=0.0900, *df*=2, *P*=0.9365<br>*t*=0.5298, *df*=2 *P*=0.6492<br>*t*=5.216, *df*=2 *P*=0.0348		 ns<br>ns<br>*
**S1A CXCL12**<br>*Grk2*$^{-/-}$<br>*Grk6*$^{-/-}$<br>2x*Grk*$^{-/-}$	One sample *t* test	 *t*=20.09, *df*=2, *P*=0.0025<br>*t*=2.642, *df*=3 *P*=0.0776<br>*t*=6.434, *df*=3 *P*=0.0076		 **<br>ns<br>**
**S1B S1P**<br>10nM<br><br>1µM	ANOVA	 *F*=2.513, *DFn*=2,<br>*DFd*=11, *P*=0.1262<br>*F*=6.337, *DFn*=2,<br>*DFd*=11, *P*=0.0148	 2x*Grk*$^{-/-}$:*Grk2*$^{-/-}$ *P*=0.3240<br>2x*Grk*$^{-/-}$:*Grk6*$^{-/-}$ *P*=0.7525<br>2x*Grk*$^{-/-}$:*Grk2*$^{-/-}$ *P*=0.1528<br>2x*Grk*$^{-/-}$:*Grk6*$^{-/-}$ *P*=0.3368	 ns<br>ns<br>ns<br>ns
**S1B CCL19**<br>0.01µg/ml<br><br>0.1µg/ml	ANOVA	 *F*=3.830, *DFn*=2, *DFd*=5,<br>*P*=0.0980<br>*F*=12.65, *DFn*=2,<br>*DFd*=12, *P*=0.0011	 2x*Grk*$^{-/-}$:*Grk2*$^{-/-}$ *P*=0.1231<br>2x*Grk*$^{-/-}$:*Grk6*$^{-/-}$ *P*=0.0713<br>2x*Grk*$^{-/-}$:*Grk2*$^{-/-}$ *P*=0.0077<br>2x*Grk*$^{-/-}$:*Grk6*$^{-/-}$ *P*=0.0010	 ns<br>ns<br>**<br>**
**S1B CXC12**	ANOVA	*F*=10.68, *DFn*=2, *DFd*=5,<br>*P*=0.0157	2x*Grk*$^{-/-}$:*Grk2*$^{-/-}$ *P*=0.0103<br>2x*Grk*$^{-/-}$:*Grk6*$^{-/-}$ *P*=0.1934	*<br>ns
**S1C CXCL13**<br>*Grk2*$^{-/-}$<br>*Grk6*$^{-/-}$<br>2x*Grk*$^{-/-}$	One sample *t* test	 *t*=0.3614, *df*=4, *P*=0.7361<br>*t*=0.5167, *df*=4 *P*=0.6326<br>*t*=1.898, *df*=4 *P*=0.1306		 ns<br>ns<br>ns
**S1C S1P**<br>*Grk2*$^{-/-}$<br>*Grk6*$^{-/-}$<br>2x*Grk*$^{-/-}$	One sample *t* test	 *t*=12.77, *df*=5, *P*<0.0001<br>*t*=1.108, *df*=2 *P*=0.3834<br>*t*=5.351, *df*=3 *P*=0.0128		 ***<br>ns<br>*
**S1C CCL19**<br>*Grk2*$^{-/-}$<br>*Grk6*$^{-/-}$<br>2x*Grk*$^{-/-}$	One sample *t* test	 *t*=3.770, *df*=5, *P*=0.0130<br>*t*=2.586, *df*=6 *P*=0.0414<br>*t*=23.31, *df*=3 *P*=0.0002		 *<br>*<br>***
**S1C CCL21**<br>*Grk2*$^{-/-}$<br>*Grk6*$^{-/-}$<br>2x*Grk*$^{-/-}$	One sample *t* test	 *t*=1.333, *df*=3, *P*=0.2746<br>*t*=1.069, *df*=4, *P*=0.3455<br>*t*=2.621, *df*=5, *P*=0.0470		 ns<br>ns<br>*
**S1C CXCL12**<br>*Grk2*$^{-/-}$<br>*Grk6*$^{-/-}$<br>2x*Grk*$^{-/-}$	One sample *t* test	 *t*=0.1351, *df*=2, *P*=0.9049<br>*t*=0.5078, *df*=2, *P*=0.6621<br>*t*=4.144, *df*=3, *P*=0.0255		 ns<br>ns<br>*
**S1D S1P**<br>10nM<br><br>1µM	ANOVA	 *F*=17.88, *DFn*=2, *DFd*=7,<br>*P*=0.0018<br>*F*=9.223, *DFn*=2, *DFd*=7,<br>*P*=0.0109	 2x*Grk*$^{-/-}$:*Grk2*$^{-/-}$ *P*=0.9895<br>2x*Grk*$^{-/-}$:*Grk6*$^{-/-}$ *P*=0.0020<br>2x*Grk*$^{-/-}$:*Grk2*$^{-/-}$ *P*=0.7896<br>2x*Grk*$^{-/-}$:*Grk6*$^{-/-}$ *P*=0.0084	 ns<br>**<br>ns<br>**
**S1D CCL19**<br>0.01µg/ml<br><br>1µg/ml	ANOVA	 *F*=9.135, *DFn*=2, *DFd*=9,<br>*P*=0.0068<br>*F*=7.153, *DFn*=2, *DFd*=8,<br>*P*=0.0165	 2x*Grk*$^{-/-}$:*Grk2*$^{-/-}$ *P*=0.0040<br>2x*Grk*$^{-/-}$:*Grk6*$^{-/-}$ *P*=0.2144<br>2x*Grk*$^{-/-}$:*Grk2*$^{-/-}$ *P*=0.0157<br>2x*Grk*$^{-/-}$:*Grk6*$^{-/-}$ *P*=0.9574	 **<br>ns<br>*<br>ns
**S1D CXC12**	ANOVA	*F*=3.316, *DFn*=2, *DFd*=7,<br>*P*=0.0970	2x*Grk*$^{-/-}$:*Grk2*$^{-/-}$ *P*=0.5578<br>2x*Grk*$^{-/-}$:*Grk6*$^{-/-}$ *P*=0.0659	ns<br>ns
**S1D CXCL13**	ANOVA	*F*=0.7207, *DFn*=2,<br>*DFd*=7, *P*=0.5193	2x*Grk*$^{-/-}$:*Grk2*$^{-/-}$ *P*=0.9983<br>2x*Grk*$^{-/-}$:*Grk6*$^{-/-}$ *P*=0.4655	ns<br>ns

**S1E**	ANOVA	F=1.108, DFn=3,<br>DFd=60, P=0.3531	WT:2x$Grk^{-/-}$ P=0.8449<br>WT:$Grk2^{-/-}$ P=0.4384<br>WT:$Grk6^{-/-}$ P=0.8546	ns<br>ns<br>ns
**S1F CD18**<br>**Fo. B cells**	ANOVA	F=0.8033, DFn=3,<br>DFd=18, P=0.5082	WT:$Grk2^{-/-}$ P=0.7492<br>WT:$Grk6^{-/-}$ P=0.4301<br>WT:2x$Grk^{-/-}$ P=0.7712	ns<br>ns<br>ns
**S1F CD18**<br>**MZ B cells**	ANOVA	F=3.920, DFn=3,<br>DFd=18, P=0.0257	WT:$Grk2^{-/-}$ P=0.7375<br>WT:$Grk6^{-/-}$ P=0.0597<br>WT:2x$Grk^{-/-}$ P=0.1877	ns<br>ns<br>ns
**S1F CD29**<br>**Fo. B cells**	Kruskal-Wallis	H(4)=10.78, P=0.0130	WT:$Grk2^{-/-}$ P=0.0062<br>WT:$Grk6^{-/-}$ P=0.3421<br>WT:2x$Grk^{-/-}$ P=0.2884	**<br>ns<br>ns
**S1F CD29**<br>**MZ B cells**	Kruskal-Wallis	H(4)=8.381, P=0.0388	WT:$Grk2^{-/-}$ P=0.0229<br>WT:$Grk6^{-/-}$ P=0.3970<br>WT:2x$Grk^{-/-}$ P>0.9999	*<br>ns<br>ns
**S1G VCAM-1**<br>*Fo Grk2*$^{-/-}$<br>*Fo Grk6*$^{-/-}$<br>Fo 2x$Grk^{-/-}$<br>*MZ Grk2*$^{-/-}$<br>*MZ Grk6*$^{-/-}$<br>MZ 2x$Grk^{-/-}$	One sample *t* test	 t=1.034, df=2, P=0.4097<br>t=0.9404, df=2, P=0.4463<br>t=0.2852, df=2, P=0.8023<br>t=1.764, df=2, P=0.2197<br>t=0.9749, df=2, P=0.4324<br>t=0.2578, df=2, P=0.8207		 ns<br>ns<br>ns<br>ns<br>ns<br>ns
**S1G ICAM-1**<br>*Fo Grk2*$^{-/-}$<br>*Fo Grk6*$^{-/-}$<br>Fo 2x$Grk^{-/-}$<br>*MZ Grk2*$^{-/-}$<br>*MZ Grk6*$^{-/-}$<br>MZ 2x$Grk^{-/-}$	One sample *t* test	 t=0.7606, df=2, P=0.5264<br>t=0.3865, df=2, P=0.7364<br>t=2.717, df=2, P=0.1130<br>t=0.1283, df=2, P=0.9096<br>t=0.9404, df=2, P=0.4463<br>t=1.627, df=2, P=0.2452		 ns<br>ns<br>ns<br>ns<br>ns<br>ns
**S2B**	Kruskal-Wallis	H(5)=19.22, P=0.0007	WT:3x$Grk^{-/-}$ P>0.9999<br>WT:$Grk3^{-/-}$ P>0.9999<br>WT:$Grk6^{-/-}$ P=0.0120<br>WT:2x$Grk^{-/-}$ P=0.0549	ns<br>ns<br>*<br>ns
**S2E**	ANOVA	F=3.621, DFn=3,<br>DFd=248, P=0.0138	earlyWT:early3x$Grk^{-/-}$$P$=0.1425<br>lateWT:late3x$Grk^{-/-}$ P=0.3751<br>earlyWT:lateWT P=0.3393	ns<br>ns<br>ns
**S3A**<br>Total cells<br>CD11b^{+}<br>CD11b^{-}	 *t* test<br>Mann-Whitney<br>*t* test	 t=2.822, df=6, P=0.0303<br>U=2, n=24,12, P=0.1143<br>t=3.756, df=6, P=0.0094		 *<br>ns<br>**
**S3B**<br>GMP<br>Monos<br>CMP&MDP<br>cMoP&GP<br>CDP	One sample *t* test	 t=2.599, df=3, P=0.0805<br>t=0.5973, df=3, P=0.5924<br>t=4.827, df=3, P=0.0169<br>t=0.5817, df=3, P=0.6016<br>t=7.328, df=3, P=0.0052		 ns<br>ns<br>*<br>ns<br>**
**S3C**<br>GMP<br>Monos<br>CMP&MDP<br>cMoP&GP<br>CDP	One sample *t* test	 t=0.2962, df=3, P=0.7864<br>t=0.1794, df=3, P=0.8691<br>t=0.2751, df=3, P=0.8011<br>t=1.272, df=3, P=0.2931<br>t=0.9115 df=3, P=0.4292		 ns<br>ns<br>ns<br>ns<br>ns
**S3D**	One sample *t* test	t=10.22, df=22, P<0.0001		***
**S3E**<br>CD11c	One sample *t* test	 t=2.834, df=2, P=0.1052		 ns

CCR7 surface<br>CCR7 total<br>MHCII<br>CD80<br>CD86<br>CD40		*t*=0.8955, *df*=2, *P*=0.4650<br>*t*=2.390, *df*=2, *P*=0.1394<br>*t*=2.442, *df*=2, *P*=0.1346<br>*t*=3.313, *df*=2, *P*=0.0803<br>*t*=2.081, *df*=2, *P*=0.1729<br>*t*=2.196, *df*=2, *P*=0.1593		ns<br>ns<br>ns<br>ns<br>ns<br>ns
**S3F**<br>DC unstim<br>Mac unstim<br>DC stim<br>Mac unstim	One sample *t* test	 *t*=1.509, *df*=3, *P*=0.2285<br>*t*=2.294, *df*=4, *P*=0.0835<br>*t*=0.3550, *df*=4, *P*=0.7405<br>*t*=3.553, *df*=5, *P*=0.0163		 ns<br>ns<br>ns<br>*
**S4A**<br>Spleen<br>LN	One sample *t* test	 *t*=2.052, *df*=15, *P*=0.0581<br>*t*=4.650, *df*=11, *P*=0.0007		 ns<br>***
